# Eosinophils and IL-4 Support Nematode Growth Coincident with an Innate Response to Tissue Injury

**DOI:** 10.1371/journal.ppat.1005347

**Published:** 2015-12-31

**Authors:** Lu Huang, Daniel P. Beiting, Nebiat G. Gebreselassie, Lucille F. Gagliardo, Maura C. Ruyechan, Nancy A. Lee, James J. Lee, Judith A. Appleton

**Affiliations:** 1 Baker Institute for Animal Health, College of Veterinary Medicine, Cornell University, Ithaca, New York, United States of America; 2 Department of Pathobiology, School of Veterinary Medicine, University of Pennsylvania, Philadelphia, Pennsylvania, United States of America; 3 Department of Biochemistry and Molecular Biology, Division of Hematology/Oncology, Mayo Clinic Arizona, Scottsdale, Arizona, United States of America; 4 Department of Biochemistry and Molecular Biology, Division of Pulmonary Medicine, Mayo Clinic Arizona, Scottsdale, Arizona, United States of America; University of York, UNITED KINGDOM

## Abstract

It has become increasingly clear that the functions of eosinophils extend beyond host defense and allergy to metabolism and tissue regeneration. These influences have strong potential to be relevant in worm infections in which eosinophils are prominent and parasites rely on the host for nutrients to support growth or reproduction. The aim of this study was to investigate the mechanism underlying the observation that eosinophils promote growth of *Trichinella spiralis* larvae in skeletal muscle. Our results indicate that IL-4 and eosinophils are necessary for normal larval growth and that eosinophils from IL-4 competent mice are sufficient to support growth. The eosinophil-mediated effect operates in the absence of adaptive immunity. Following invasion by newborn larvae, host gene expression in skeletal muscle was compatible with a regenerative response and a shift in the source of energy in infected tissue. The presence of eosinophils suppressed local inflammation while also influencing nutrient homeostasis in muscle. Redistribution of glucose transporter 4 (GLUT4) and phosphorylation of Akt were observed in nurse cells, consistent with enhancement of glucose uptake and glycogen storage by larvae that is known to occur. The data are consistent with a mechanism in which eosinophils promote larval growth by an IL-4 dependent mechanism that limits local interferon-driven responses that otherwise alter nutrient metabolism in infected muscle. Our findings document a novel interaction between parasite and host in which worms have evolved a strategy to co-opt an innate host cell response in a way that facilitates their growth.

## Author Summary

Eosinophilia is a central feature of Type 2 immunity induced by infection with parasitic worms. Although early work showed that eosinophils could adhere to and damage parasite larvae *in vitro*, a definitive role for eosinophils during worm infection remained elusive for many years. Recent studies uncovered novel roles of eosinophils in regulating metabolism and tissue remodeling, observations that suggest that eosinophils may function as regulatory cells and modulate such processes during helminth infections. We investigated the eosinophil-dependent mechanism that promotes growth of *Trichinella spiralis* larvae. We found that larval growth is independent of adaptive immunity and requires IL-4/STAT6 signaling in eosinophils. Gene transcription profiles in infected muscle suggested that eosinophils promote larval growth by suppressing local inflammation and enhancing nutrient uptake and metabolism. Our study provides new insights into the interactions between a parasitic worm and its host.

## Introduction

Genomic analysis of diverse members of the Nematoda has revealed that parasitism emerged in free-living nematodes as a result of multiple, independent evolutionary events [1]. The process of host adaptation is associated with loss of functions that enable a free-living lifestyle. Understanding these dependencies is instructive when devising therapeutic and prophylactic approaches to controlling parasitic disease.

A central influence on successful parasitism is the host immune response. Animals often mount highly effective Th2 immune responses against worms; however, specific effector mechanisms vary by host, tissue and parasite. As counterpoint, helminths co-opt or evade the host immune response in ways that ensure completion of life cycle and transmission to the next host. In two examples, schistosomes manipulate innate immune signals and Th2 immunity to facilitate their development [2,3] and *Trichinella* reproduces in the host intestine within 5 days of infection, in advance of a potent intestinal immune response that expels adult worms [4].

Eosinophilia is a hallmark of the host immune response to parasitic worms. Long considered to be cytotoxic effector cells that kill parasite larvae, recent studies in eosinophil-ablated mice have shown that the influence of eosinophils on worms varies from no effect, to protecting the host, to supporting the parasite [5–9]. In separate research on animal metabolism, new findings document a regulatory influence of eosinophils [10–13]. Control of energy metabolism by eosinophils has potential to influence the outcome of worm infection.

After being released from female adult worms in the intestine, *Trichinella spiralis (T*. *spiralis)* newborn larvae (NBL) enter the circulatory system, migrate to a variety of tissues and eventually establish intracellular infection in skeletal muscle. Following invasion of skeletal muscle cells, NBL remain inactive for 4 days prior to initiating growth from 130 μm to 1 mm in length over the course of 20 days [14]. Our previous studies demonstrated that eosinophils are recruited to muscle when NBL arrive and that they positively regulate both survival and growth of larvae [5,7,9]. Larval survival is dependent on eosinophil-derived interleukin (IL)-10. This, in turn, drives production of CD4^+^IL-10^+^ cells that suppress local nitric oxide (NO) production by neutrophils and macrophages [9]. NO is toxic for newborn and growing larvae [5,9].

A distinct mechanism that is independent of IL-10 [5] supports growth of *T*. *spiralis* larvae. This was the focus of the current study. Our results reveal a requirement for IL-4/signal transducers and activators of transcription 6 (STAT6) signaling in eosinophils for normal larval growth. In the absence of eosinophils, there is a sustained, local, interferon-driven inflammation that promotes a shift in metabolism in infected muscle. These findings provide evidence for a host-parasite relationship in which the recruitment of eosinophils promotes a nutrient-rich environment that facilitates larval growth.

## Results

### Larval growth is regulated by IL-4/STAT6 signaling pathway

**Fig 1:**
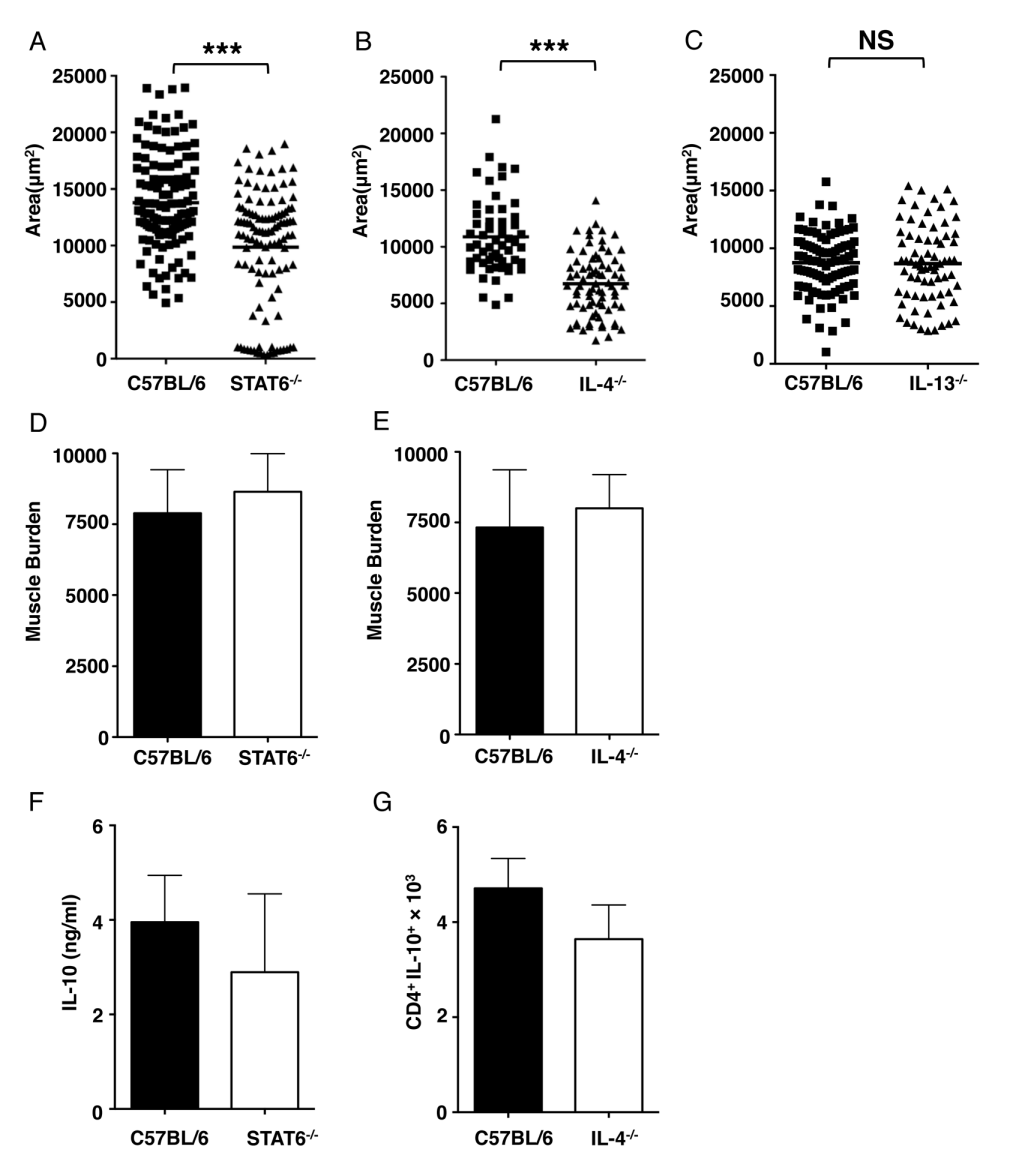
IL-4/STAT6 signaling pathway is essential for larval growth. Area of larvae recovered from (A)–(C), C57BL/6, STAT6^-/-^, IL-4^-/-^ and IL-13^-/-^ mice injected 25,000 NBL IV, 13 days post injection. Total body larval burdens in muscles of (D) WT and STAT6^-/-^ and (E) WT and IL-4^-/-^, 24 days post injection. (F) IL-10 detected in CLN cultures from WT and STAT6^-/-^ mice, 13 days post injection. (G) Number of CD4^+^IL-10^+^ cells per diaphragm of WT and IL-4^-/-^ mice, 13 days post injection. Each data set was collected from two experiments with similar results. Values represent mean ± SD; n = 4 mice. Significant differences were determined by Student’s *t* test. ***p < 0.0001.

Our previous studies showed that reduced numbers of Th2 cells in *T*. *spiralis* infected muscle were associated with compromised larval growth in eosinophil-ablated mice. To further dissect the interaction between Th2 immunity, larval growth, and larval development, we synchronously infected STAT6^-/-^ mice with NBL. Larval growth was impaired in the absence of STAT6 (Fig 1A); however, neither larval survival nor IL-10 responses in CLN cells was affected (Fig 1D and 1F), suggesting that STAT6 signaling is essential for parasite growth but not required for the IL-10 dependent protection of larvae from NO-mediated killing [5,7,9]. This result complements our earlier finding that larvae grow normally in IL-10^-/-^ mice but are eventually killed by poorly controlled NO [5]. The dependence of growth on STAT6 further distinguishes the mechanisms underlying eosinophil-dependent growth versus protection from NO.

IL-4 and IL-13 signal via the STAT6 pathway. Growth of larvae in intravenously (IV) infected IL-4^-/-^ mice was impaired while both larval survival and the number of CD4^+^IL-10^+^ cells in diaphragms were similar to wild type (WT) mice (Fig 1B, 1E and 1G). In contrast, IV infected IL-13^-/-^ mice supported larval growth normally (Fig 1C). Although the mean area of larvae from WT mice in Fig 1C is lower than that of WT mice in Fig 1A and 1B, a replicate experiment in IL-13^-/-^ mice confirmed that growth was not impaired in the absence of IL-13 (WT, 7495 ± 327 μm^2^ versus IL-13^-/-^, 7073 ± 358 μm^2^). The results provide further evidence that larval growth and survival are controlled by distinct mechanisms. Furthermore, IL-4 and STAT6 are essential for larval growth, while IL-13 is dispensable.

### STAT6 signaling in bone marrow-derived cells regulates larval growth

**Fig 2:**
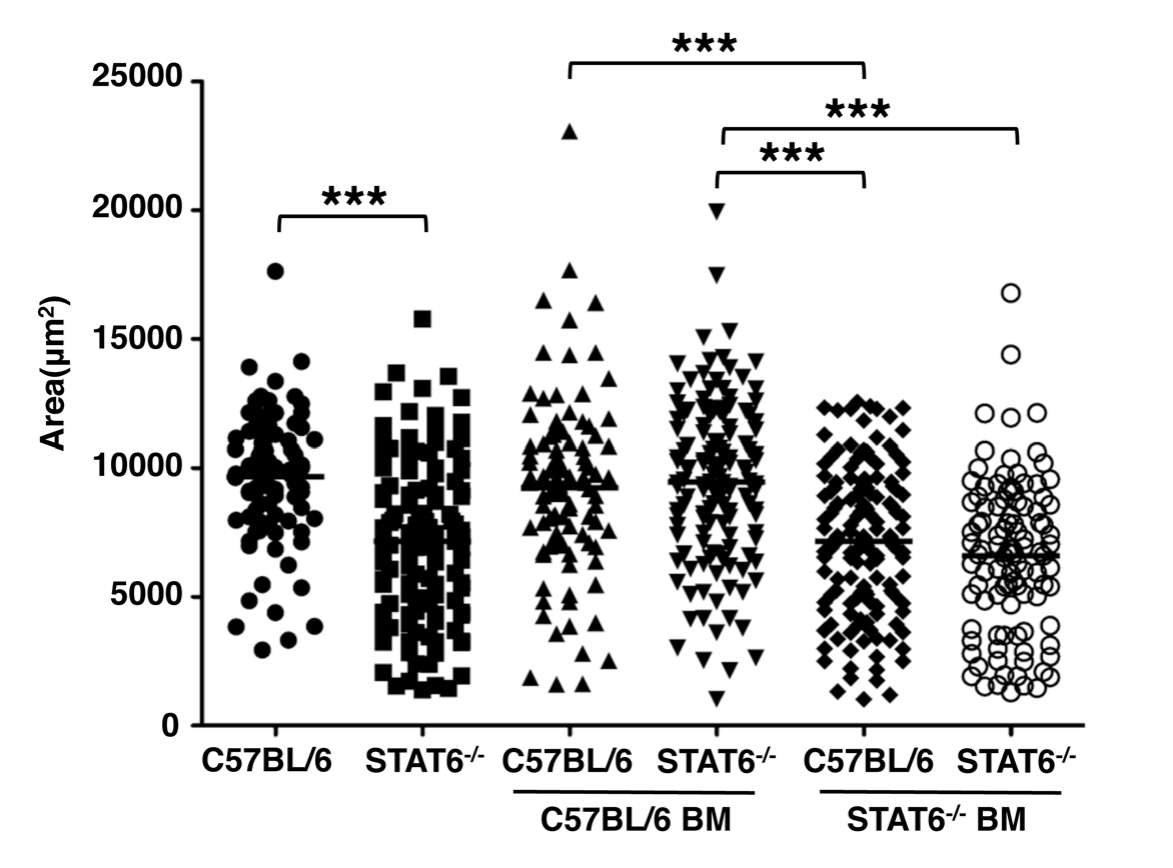
STAT6 signaling in bone marrow-derived cells is essential for parasite growth. B6.SJL and STAT6^-/-^ mice were reconstituted with bone marrow cells from B6.SJL or STAT6^-/-^ mice for eight weeks, then infected by IV injection of 25,000 NBL. Areas of larvae were estimated, 13 days post injection. Each data set was collected from two experiments with similar results. n = 4 mice. Significant differences were determined by ANOVA and Tukey’s test. ***p < 0.0001.

To further examine STAT6-dependent larval growth, we set out to determine whether IL-4 may be signaling via STAT6 in skeletal muscle cells. We infected bone marrow chimeric mice prepared from B6.SJL and STAT6^-/-^ mice. Only mice with STAT6 competent bone marrow were able to support larval growth (Fig 2).

### IL-4/STAT6 signaling in eosinophils promotes larval growth

**Fig 3:**
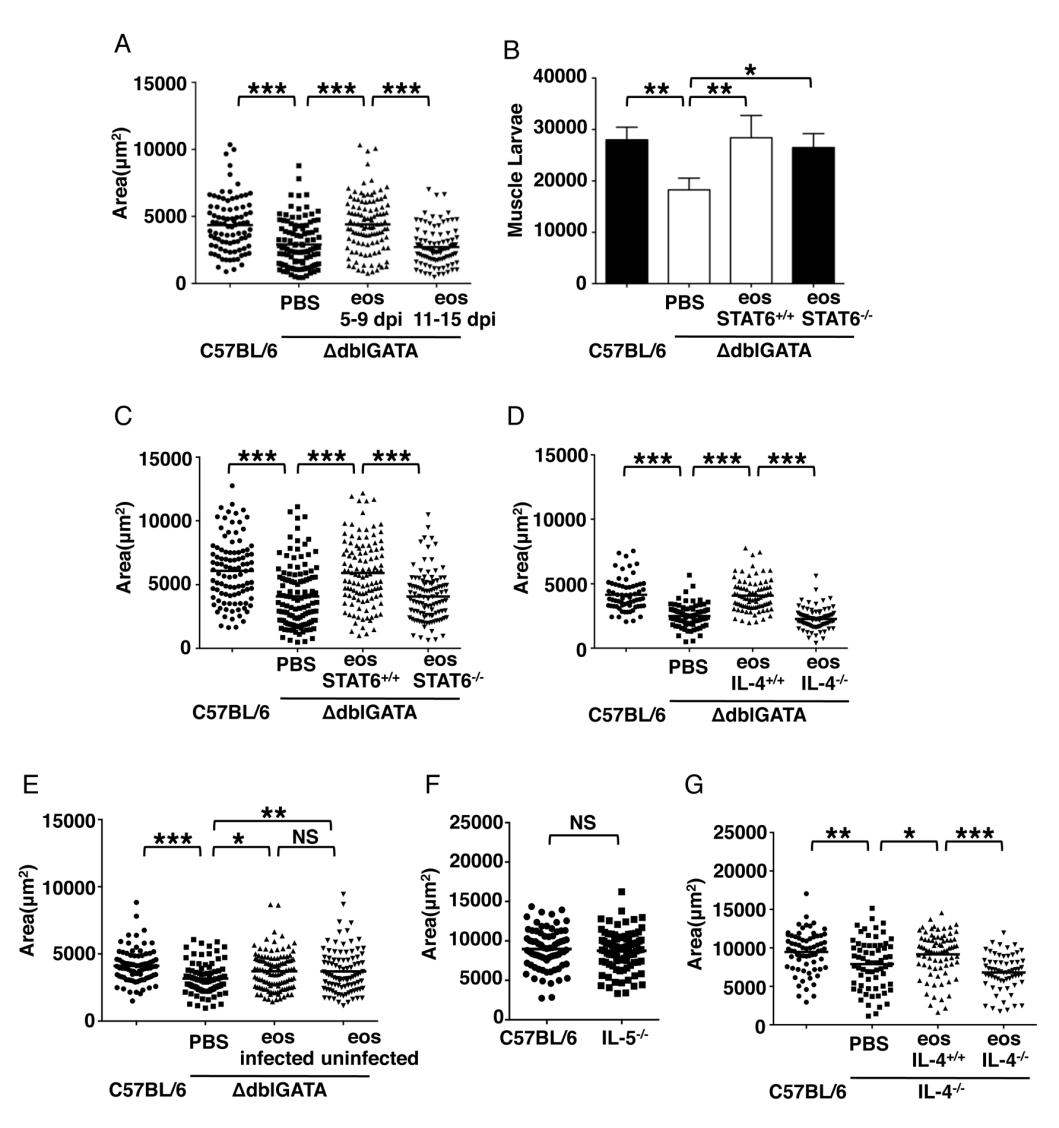
IL-4/STAT6 signaling in eosinophils is required for larval growth. (A) Body size (area) of larvae, 17dpi. ΔdblGATA mice received PBS or 5 × 10^6^ eosinophils every 48 h between 5 and 9 dpi or 11 and 15 dpi (oral infection). (B)–(C), ΔdblGATA mice received PBS or 5 × 10^6^ eosinophils from infected IL-5Tg^+^ or IL-5Tg/STAT6^-/-^ mice every 48 h from 5–9 dpi. (B) Total body larval burdens in muscle, 28 dpi. (C) Body size (area) of larvae, 17 dpi. (D) ΔdblGATA mice received PBS or 5 × 10^6^ eosinophils from infected IL-5Tg^+^ or IL-5Tg^+^/IL-4^-/-^ mice every 48 h from 5–9 dpi. Body size (area) of larvae, 17dpi. (E) ΔdblGATA mice received PBS or 5 × 10^6^ eosinophils from uninfected or infected IL-5Tg^+^ every 48 h from 5–9 dpi. Body size (area) of larvae, 17dpi. (F) Body size (area) of larvae recovered from C57BL/6 and IL-5^-/-^ mice injected 25,000 NBL IV, 13 days post injection. (G) IL-4^-/-^ mice received PBS or 5 × 10^6^ eosinophils from infected IL-5Tg^+^ or IL-5Tg^+^/IL-4^-/-^ mice every 48 h from 0–5 days post IV infection. Body size (area) of larvae, 13 days post injection. Each data set was collected from two experiments with similar results. Values represent mean ± SD; n = 4 mice. Significant differences were determined by ANOVA and Tukey’s test. *p < 0.05, **p < 0.001, ***p < 0.0001.

We next determined the time during infection when eosinophils influence larval growth. Transfer of eosinophils to infected ΔdblGATA mice during two different intervals confirmed that larval growth was supported when eosinophils were transferred between days 5 and 9 post oral infection of recipients. In contrast, transfer between days 11 and 15 had no effect on larval growth (Fig 3A). Thus, normal growth of larvae required eosinophils to be available at a time coincident with, or immediately following arrival of larvae in muscle. In similar transfer experiments, survival of larvae was not dependent upon STAT6 in eosinophils (Fig 3B); however, only recipients of STAT6^+/+^ eosinophils supported larval growth (Fig 3C). Similarly, transfer of IL-4^-/-^ eosinophils isolated from infected IL-5Tg^+^/IL-4^-/-^ mice did not improve larval growth in ΔdblGATA mice (Fig 3D). Eosinophils isolated from uninfected IL-5Tg^+^ were as effective as those isolated from infected IL-5Tg^+^ mice in restoring larval growth in ΔdblGATA mice (Fig 3E). Thus, infection and the resulting immune response are not necessary for conditioning the eosinophil prior to transfer. Furthermore, growth of larvae in IL-5^-/-^ infected mice was not impaired (Fig 3F). Thus, IL-5 is not required for the growth-supporting properties of eosinophils. Lastly, to test whether eosinophils were driving an amplified IL-4 response in recipient mice that in turn supported larval growth, eosinophils isolated from infected IL-5Tg^+^ mice were transferred to infected IL-4^-/-^ mice. Improved larval growth was observed only in recipients of IL-4^+/+^ eosinophils (Fig 3G). Thus, larval growth requires only that eosinophils be derived from IL-4 competent donors and the mechanism does involve amplification of IL-4 production by other cells during infection.

### Eosinophils support larval growth independently of adaptive immunity

**Fig 4:**
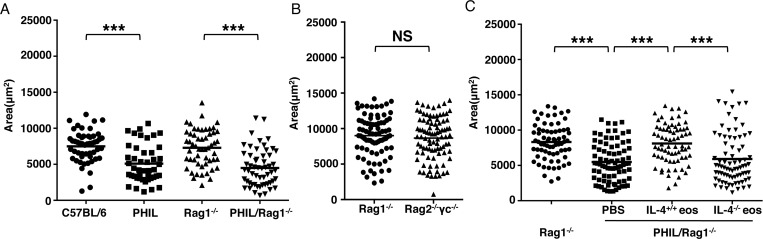
Eosinophils promote larval growth in an innate context. (A)–(C), C57BL/6, PHIL, Rag1^-/-^, PHIL/Rag1^-/-^ and Rag2^-/-^γc^-/-^ mice were injected IV with 25,000 NBL. (A) Body size (area) of larvae recovered from indicated strains 13 days post injection. (B) Body size (area) of larvae recovered from Rag1^-/-^ and Rag2^-/-^γc^-/-^ mice, 13 days post injection. (C) PHIL/Rag1^-/-^ mice received PBS or 5 × 10^6^ eosinophils from infected IL-5Tg^+^ or IL-5Tg^+^/IL-4^-/-^ mice every 48 h from 0–5 days post IV infection. Body size (area) of larvae, 13 days post injection. Each data set was collected from two experiments with similar results. Values represent mean ± SD; n = 3–4 mice. Significant differences were determined by Student’s *t* test or ANOVA and Tukey’s test. ***p < 0.0001.

We next tested the requirement for adaptive immunity in larval growth by measuring larvae in Rag1^-/-^ mice. Notably, the morphology of nurse cells in Rag1^-/-^ mice is comparable to those in WT mice. Larvae grew normally in Rag1^-/-^ mice, while larval growth was impaired in PHIL/Rag1^-/-^ mice (Fig 4A), indicating that eosinophils promote larval growth in an innate context. Type 2 innate lymphoid cells (ILC2) promote recruitment and activation of eosinophils. To investigate whether ILC2 contributed to larval growth by recruiting and activating eosinophils, we infected Rag2^-/-^γc^-/-^ mice. Larval growth was not affected by the absence of ILC2 (Fig 4B), a result that is compatible with our previous finding that recruitment of eosinophils to infected muscle was normal in ILC2-ablated Rag2^-/-^γc^-/-^ mice [9]. Lastly, transfer of IL-4^-/-^ eosinophils failed to improve larval growth in PHIL/Rag1^-/-^ mice (Fig 4C). Taken together, the findings show that eosinophils regulated larval growth in an innate context that was independent of ILC2. The effect depended upon eosinophils being derived from IL-4 competent mice.

### NBL infection triggers reprogramming of gene transcription in muscle

**Fig 5:**
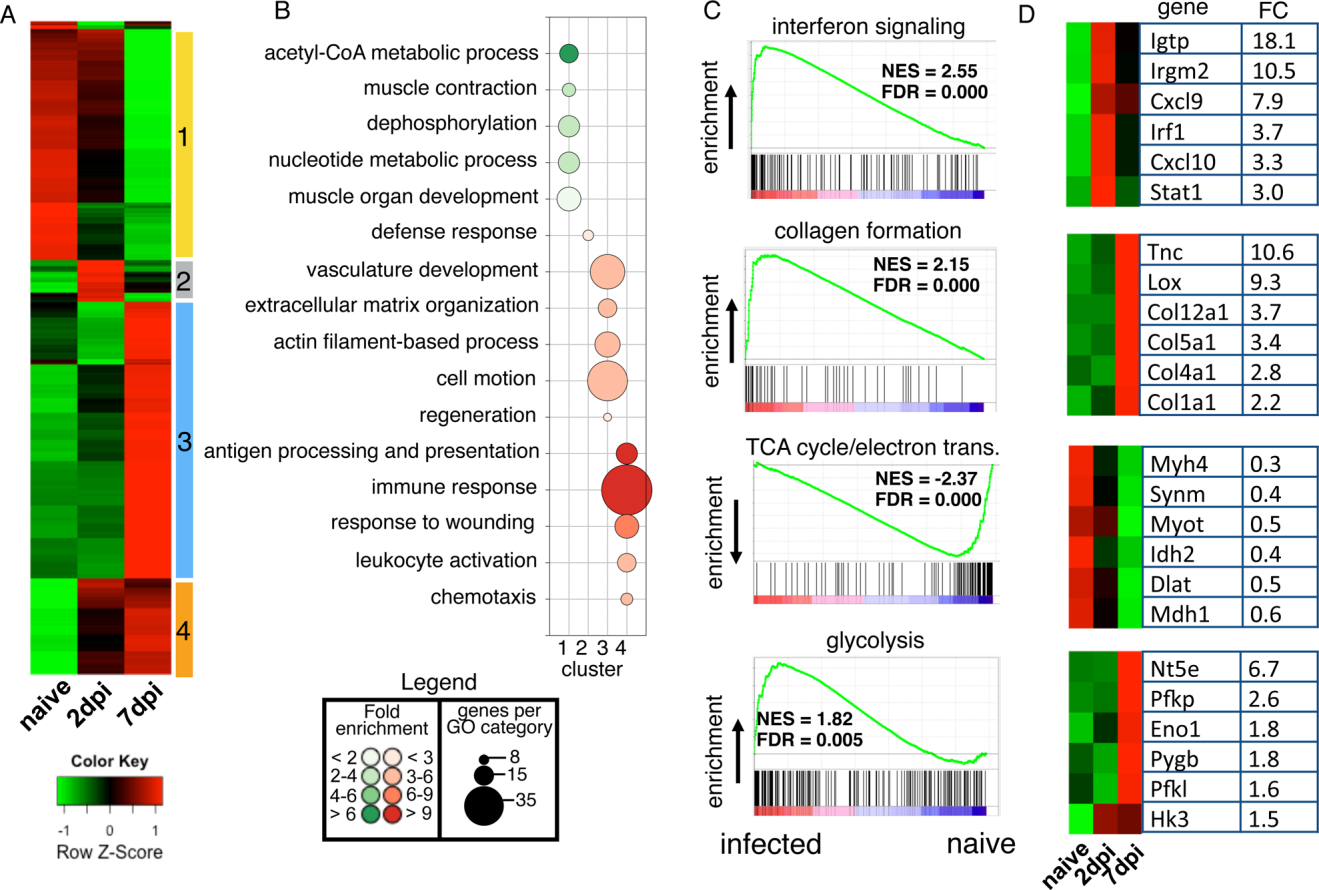
Transcriptional reprogramming of skeletal muscle during *T*. *spiralis* infection in Rag1^-/-^ mice. Microarray-based gene expression profiling of diaphragms of uninfected and infected Rag1^-/-^ mice on 0, 2 and 7 post IV infection. (A) Hierarchical clustering and heatmap representation of 1529 genes differentially regulated during a time course of *T*. *spirals* muscle infection (2-fold, FDR ≤ 0.05). Four clusters of coordinately regulated genes are indicated. Color pattern represents row Z-score. (B) Bubble chart showing results of Gene Ontology (GO) enrichment analysis. Bubble size indicates number of genes associated with each term. Bubble color indicates whether genes associated with each term were upregulated (red) or downregulated (green), while color intensity indicates fold enrichment. (C) GSEA enrichment plot for four selected pathways with normalized enrichment scores (NES) and false discover rates (FDR). Collagen formation, TCA cycle/electron trans. and glycolysis: 7dpi vs naïve. Interferon signaling: 2dpi vs naïve. (D) Six selected genes from each GSEA signature panel (C). Maximum mean fold change (FC) between naïve and infected Rag1^-/-^ mice is shown.

To further investigate the mechanism of eosinophil-mediated larval growth in skeletal muscle, we performed whole genome transcriptional profiling to identify genes altered early in the course of muscle infection, coincident with the interval during which eosinophils are required. In order to limit the numbers of infiltrating cells in muscle tissue, experiments were performed with Rag1^-/-^ mice. Muscle infection in Rag1^-/-^ mice resulted in differential expression of 1529 genes (S1 Table). Hierarchical clustering revealed at least four distinct clusters of co-regulated genes (Fig 5A). Genes in cluster 1 are repressed early during infection, with the greatest effect evident on 7 days post infection (dpi). Gene Ontology enrichment analysis revealed that this cluster included genes involved in muscle organ development and acetyl-CoA metabolism (Fig 5B). Cluster 2 is an early response profile that incorporates genes whose expression peaks at 2dpi. This gene set was highly enriched for genes in the defense response (Fig 5B). In contrast, cluster 3 includes genes that are strongly induced at 7dpi and largely involved in cellular remodeling and wound healing (Fig 5B). Finally, genes in cluster 4 are induced at 2dpi and further increased at 7dpi. This cluster included genes involved in antigen presentation and immune activation (Fig 5B). To identify pathways enriched in infected muscle, relative to uninfected muscle, gene set enrichment analysis (GSEA) was performed on 2 and 7 dpi samples. At 2dpi, there was marked induction of genes involved in interferon signaling (Fig 5C), including the STAT1 target genes Igtp, Irgm2, Cxcl9 and Cxcl10, as well as master regulators of the interferon response, Irf1 and Stat1 (Fig 5D). GSEA at 7dpi identified signatures primarily associated with collagen formation and muscle repair (Fig 5C). This included tenascin C (Tnc), a tissue remodeling factor; lysyl oxidase (Lox), a secreted enzyme that initiates crosslinking of collagen and elastin; and a series of collagen genes (Col12a1, Col5a1, Col4a1 and Col1a1) (Fig 5D). These finding were consistent with substantial muscle cell hypertrophy and initiation of nurse cell transformation and formation of the collagen capsule at this time. Notably, many genes of glycolysis were significantly induced at 7dpi, including several key regulatory enzymes such as Pfkp, Pygb, Pfkl and Hk3 (Fig 5D). A large set of genes was also markedly reduced in expression by 7dpi and included signatures related to the TCA cycle (e.g. Idh2, Dlat and Mdh1) and striated muscle cell development (e.g. Myh4, Synm and Myot) (Fig 5C and 5D). Collectively, these changes in gene expression programs following infection by NBL describe a robust early induction of STAT1-dependent genes and subsequent loss of myofibers, a strong regenerative response, and a shift in the sources of energy in infected muscle.

### Evidence of enhanced glucose uptake in nurse cells

**Fig 6:**
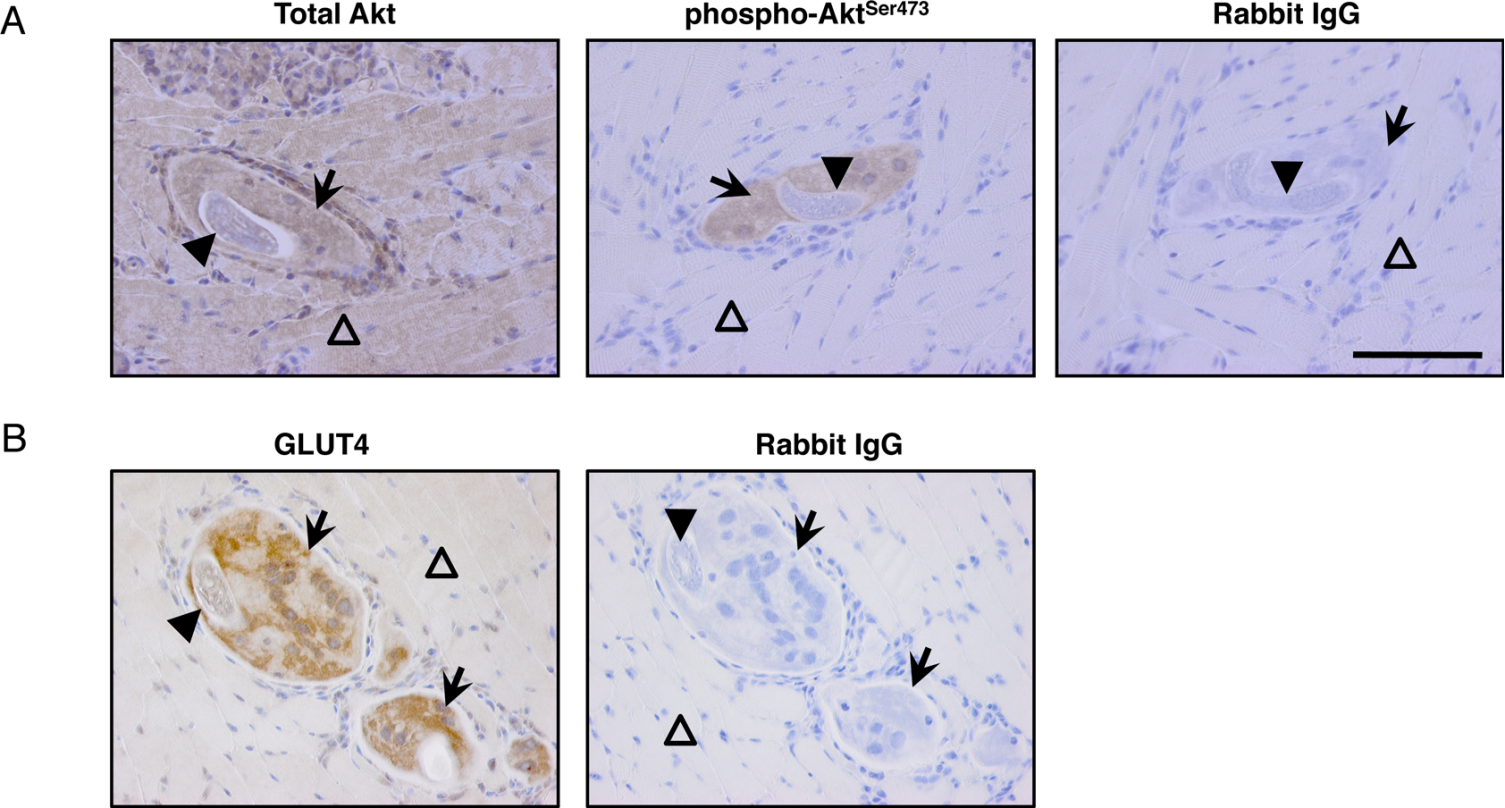
GLUT4 and phosphorylation of Akt are enhanced in nurse cells. Detection of (A) Akt, phospho-Akt^Ser473^ and (B) GLUT4 in tongues collected from Rag1^-/-^ mice infected by IV injection with NBL, 13 days post injection. Arrows indicate nurse cells. Solid arrowheads indicate muscle larvae. Opened arrowheads indicate uninfected muscle cells. Scale bar = 100μm. n = 3 mice.

The reduced expression of genes involved in acetyl-CoA metabolism suggested a change in the source of energy in infected muscle. A previous report described enhanced uptake of glucose by isolated nurse cells [15]. We detected Akt in a variety of cell types in sections of tongues collected 13 days post IV infection; however, phospho-Akt^ser473^ was specifically detected at high levels in nurse cells (Fig 6A), consistent with active glucose metabolism in nurse cells. Increased expression of Glut4 was not detected in microarray analysis; however, intracellular GLUT4 was dramatically increased and redistributed to be associated with vesicle-like structures in nurse cells, a change that is consistent with active transport of glucose (Fig 6B). These results are compatible with a shift to glucose metabolism in muscle cells during the first 7 days of infection. During this time, the nurse cell is becoming an active metabolic complex that serves as a nutrient-rich site for growing larvae that store glycogen.

### Eosinophils enhance nutrient metabolism in muscle while suppressing local inflammation

**Fig 7:**
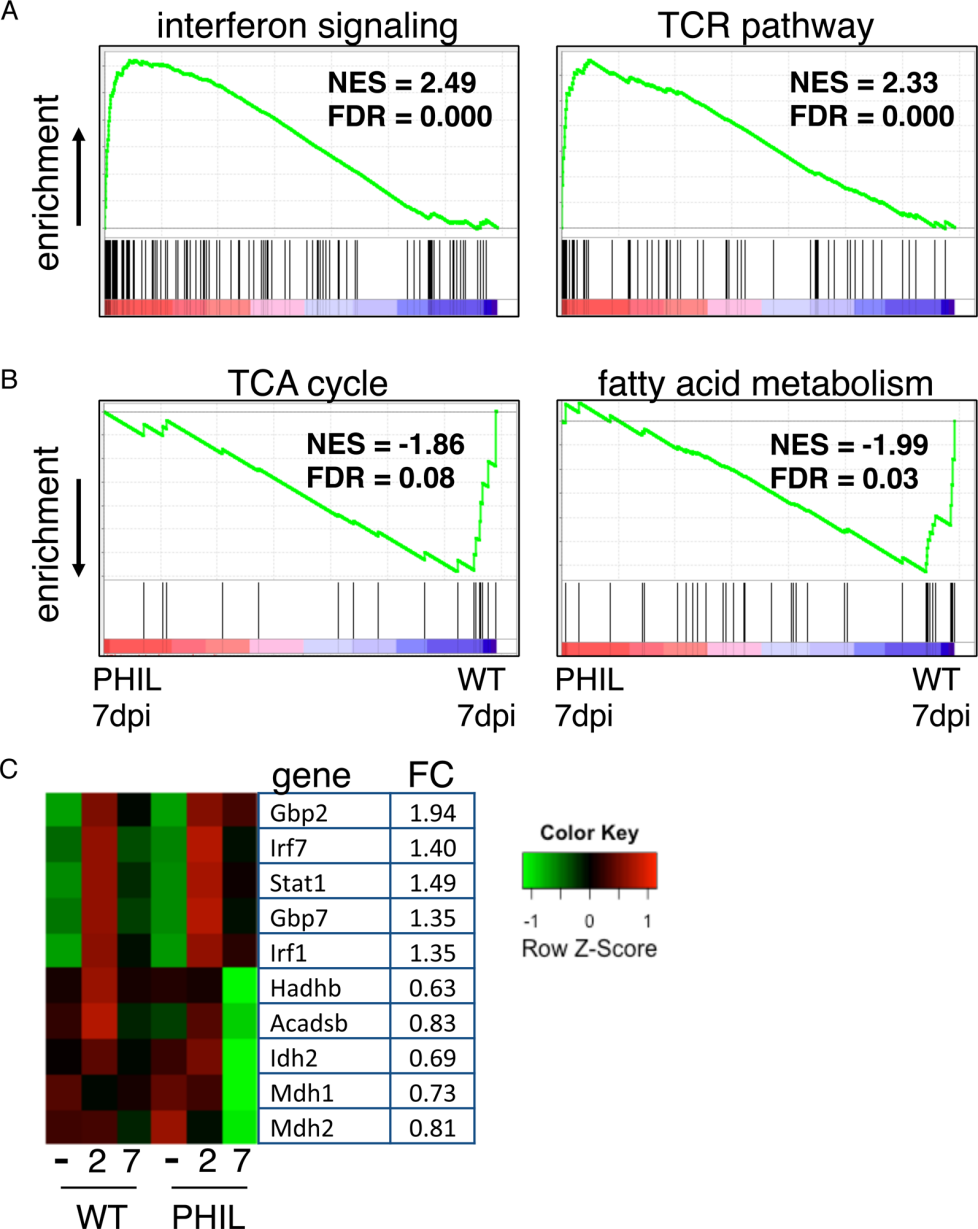
Eosinophils suppress STAT1 signaling and modulate metabolism in infected muscle. Microarray-based gene expression profiling of diaphragms of uninfected and infected WT and PHIL mice on 0, 2 and 7 days post IV infection. Pathways detected by GSEA to be enriched in (A) PHIL or (B) WT mice at 7 days post IV infection. (C) Selected genes from interferon signaling, TCA cycle and fatty acid metabolism pathways. Maximum mean fold change (FC) between PHIL and WT at 7 days post IV infection is shown. Color pattern represents row Z-score.

Given that the transcriptome analysis of tissue from infected Rag1^-/-^ mice showed a clear reprogramming of muscle, we tested a role for eosinophils in regulation by performing transcriptional analysis of infected WT and PHIL muscle at 2 and 7 dpi. Similar to Rag1^-/-^ mice, both WT and PHIL mice showed transcriptional signatures associated with a general reprogramming of muscle following infection; however there were distinct differences between WT and PHIL (S2 Table). Notably, PHIL mice exhibited sustained activation of interferon signaling genes and a STAT1 signature at 7dpi when compared to WT mice that was highly significant (Fig 7A). This gene set includes Gbp2, Gbp7, Irf7, Irf1 and Stat1 (Fig 7C). The TCR pathway was also enriched in PHIL at 7dpi (Fig 7A), reflecting the population of lymphocytes infiltrating tissue in the absence of eosinophils. Transcriptional signatures of the TCA cycle and fatty acid metabolism were reduced in PHIL mice at 7dpi compared to WT mice (Fig 7B). Genes in this set are associated with lipid metabolism (e.g. Hadhb and Acadsb) and the TCA cycle (e.g. Idh2, Mdh1 and Mdh2) (Fig 7C). Taken together, these experiments show that infection by NBL induces local inflammation and altered nutrient metabolism in skeletal muscle that is controlled by eosinophils.

## Discussion

The influence of eosinophils varies dramatically between primary and secondary infection with *T*. *spiralis* [9,16]. In secondary infection, eosinophils cooperate with antibodies to limit the number of NBL that transit the lung and colonize skeletal muscle [9,16]. During primary infection, eosinophils appear to protect the parasite rather than the host and we have reported previously that growth of *T*. *spiralis* larvae during primary infection is dependent on eosinophils [5,7,9]. Others have shown that filarial parasites regulate their development and reproduction in response to eosinophilia [17]. The dependency of worms on eosinophils for development or growth suggests a mechanism by which the host response provides resources to the parasite.

Due to the size and motility of parasitic worms, tissue injury is a feature of infection. The response of skeletal muscle to injury is regenerative. Recent findings have linked eosinophils to skeletal muscle regeneration by showing them to be the dominant IL-4 producing cells at sites of muscle damage and furthermore, that IL-4 activates regenerative responses [11]. Consistent with these findings, we found that expression of genes involved in muscle regeneration feature prominently in the response to NBL invasion and that this response is influenced by eosinophils. Our observations support a model in which eosinophils would contribute to IL-4 dependent muscle regeneration in an innate response to the injury caused by invading NBL.

Larval growth was supported when eosinophils were restored to ablated mice by transfer from IL-4 or STAT6-competent mice. Eosinophils express a functional IL-4 receptor [18] and it is possible that they must develop in the presence of IL-4 in order to produce other mediators or effector functions that support larval growth. For example, RELMα is induced in an IL-4-dependent manner in eosinophils during schistosome infection [19], and also plays critical roles in regulating host energy metabolism and glucose homeostasis [20,21]. Our microarray analysis revealed that RELMα expression increases 1.76 fold at 7 dpi compared to naïve muscle. Attribution of this change to eosinophils is not possible with the available data. The influence of RELMα and other IL-4 dependent eosinophil products on larval growth requires experimentation with gene knockouts that are inducible or otherwise targeted to eosinophils.

Alternatively, eosinophils may deliver IL-4 to other cells. Infection of STAT6 chimeras showed that when STAT6 was limited to bone marrow cells, mice supported larval growth. This eliminates the possibility that fully differentiated myotubes invaded by NBL would support growth via STAT6 signaling. It is important to note reports providing evidence that, during muscle injury and regeneration, IL-4 is capable of stimulating IL-4Rα^+^ myoblast fusion and muscle fiber growth [22]. A proportion of myoblasts arise from cells recruited from bone marrow [23–25]. Similarly, IL-4Rα^+^ fibro/adipocyte progenitors (FAP) support myogenesis [11]. Furthermore, bone marrow-derived IL-4Rα^+^ CD11b^+^ myeloid effector cells are capable of limiting excessive inflammation via IL-4 induced STAT6 signaling [26]. Any of these cells provides a target for eosinophil-derived IL-4, with potential to promote nurse cell formation, recovery from muscle injury, or control of inflammation. Transfer of IL-4 competent eosinophils to appropriately targeted IL-4 receptor knockout mice will serve to test these mechanisms.

Autophagy is another mechanism involved in maintaining skeletal muscle mass and regulating glucose homeostasis in muscle cells [27,28]. Autophagy defends cells against intracellular pathogens [29] and is inhibited by Th2 cytokines, such as IL-4 and IL-13 [29]. Furthermore, helminth products can induce autophagy [30]. After invading muscle cells, NBL establish an intracellular infection. We speculated that *T*. *spiralis* might induce the recruitment of IL-4 secreting eosinophils to sites of infection as a mechanism to prevent destruction of NBL by autophagy. Gene expression array analysis did not reveal any significant regulation of autophagy-related genes in the absence of eosinophils. Our findings do not support a role for autophagy in regulating larval growth in skeletal muscle.

*T*. *spiralis* larvae store large quantities of glycogen [31] and nurse cells transport greater quantities of glucose than skeletal muscle cells [15]. In the absence of exogenous glucose, under aerobic or anaerobic conditions, larvae rapidly break down endogenous stores of glycogen [32]. Importantly, reduced larval glycogen correlates with impaired infectivity [33]. Consistent with a deficiency of larval glycogen, surviving larvae in eosinophil-ablated mice are less infectious than larvae from WT mice [7]. Other reports show that infection by *T*. *spiralis* induces host hypoglycemia and suggest that this is the result of an increase in glucose uptake by infected muscle cells via up-regulation of the insulin signaling pathway [34]. Recent reports demonstrated that IL-4 producing eosinophils are capable of promoting insulin sensitivity in adipose tissues [10,13]. In skeletal muscle, the binding of IL-4 to its receptor activates the PI3K/Akt pathway and further promotes insulin stimulated glucose uptake via GLUT4 translocation [35]. Our bone marrow chimera experiments suggested that STAT6 in established myotubes is dispensable for larval growth; however, modification of the myotube by infiltrating cells from bone marrow may enable STAT6 signaling in the nurse cell. Nevertheless, this mechanism need not come into play, as IL-4 also engages insulin receptor substrate 2 (IRS2)/PI3K signaling to mediate Akt activation [36,37]. We found evidence for pathway activation and GLUT4 translocation in infected muscle cells, which supports a mechanism in which eosinophil-derived IL-4 promotes muscle larval growth directly by enhancing glucose uptake in host cells. Establishing this pathway as a mechanism that supports larval growth requires further experimentation.

Alternatively, glucose uptake can be regulated indirectly by IL-4. Inflammation attenuates insulin sensitivity in multiple tissues [38,39], More specifically, IFN-γ attenuates insulin sensitivity via sustained activation of the STAT1 pathway [40]. Gene expression data revealed activation of the STAT1 pathway in infected muscle (Cluster 2 in Fig 5), a profile that was sustained in eosinophil-ablated mice compared to WT (Fig 7). Thus, in the absence of eosinophils, sustained STAT1 activation may limit glucose uptake in nurse cells. Transcriptional signatures of both the TCA cycle and fatty acid metabolism are dramatically reduced in the absence of eosinophils. IL-4 has been shown to be important for promoting lipolysis and glucose homeostasis [41,42]. In aggregate, the gene expression data suggest that eosinophils contribute to the maintenance of glucose and lipid metabolism in muscle at an early stage of larval infection.

Our results are consistent with a mechanism in which larval growth is supported when eosinophils, or eosinophil-derived IL-4, limits STAT1 signaling that otherwise would inhibit glucose and lipid metabolism in muscle. Further experimentation with gene knockouts targeted to eosinophils, or the cells they influence, will be important to clarifying the mechanism. Our results document a process by which a parasitic worm appears to co-opt innate responses to injury in a way that supports its life cycle. Eosinophils are central to this process and operate in the context of innate immunity.

## Materials and Methods

### Ethics statement

Animal procedures were performed in strict accordance with the recommendations in the Guide for the Care and Use of Laboratory Animals of the National Institutes of Health. The protocol was approved by the Institutional Animal Care and Use Committee of Cornell University (Protocol number: 1986–0058).

### Rats and mice

Adult Albino Oxford strain rats were produced and maintained in the Baker Institute vivarium. PHIL (eosinophil-ablated), ΔdblGATA (eosinophil-ablated), Rag1^-/-^(T and B cell-ablated), PHIL/Rag1^-/-^ and IL-5-expressing transgenic (NJ.1638)(IL-5Tg^+^), IL-5Tg^+^/ IL-4^-/-^, IL-5Tg^+^ /STAT6^-/-^ and IL-5^-/-^ mice were bred at Cornell Transgenic Mouse Core Facility (TMCF) and offspring were transferred to the Baker Institute. IL-4^-/-^ and STAT6^-/-^ mice were purchased from The Jackson Laboratory and the bred at TMCF.

PHIL/Rag1^-/-^, IL-5Tg^+^/IL-4^-/-^ and IL-5Tg^+^/STAT6^-/-^ mice were generated by crossing and backcrossing on the deficient strains and genotype was confirmed by PCR. Rag2^-/-^γc^-/-^ (innate lymphoid cell-ablated) and B6.SJL mice were purchased from Taconic. IL-13^-/-^ mice were a gift from Dr. Avery August (Cornell) and Dr. Thomas Wynn (NIH). PHIL mice were genotyped as described previously [43]. All strains were on a C57BL/6 background. C57BL/6 ^NHsd^ mice were purchased from Taconic as WT control.

### Parasite and antigens

*Trichinella spiralis* first-stage larvae (L_1_) and NBL were recovered from rats as described previously [7]. For oral infection, L_1_ were suspended in 2% nutrient broth (Difco), 0.6% gelatin (Fisher Scientific) and doses of 300 L_1_ were administered by gavage. For synchronous infection, 25,000 NBL were suspended in 0.25 ml serum-free DMEM (Mediatech, Inc.) and delivered by retro-orbital injection. Mice were euthanized by CO_2_ inhalation at the times indicated in each experiment. Whole body muscle larvae burdens were assessed in carcasses 28 days post oral infection or 24 days post NBL injection as described previously [7]. Crude somatic antigens from L_1_ were prepared as previously described [7].

### Parasite measurement

Area of parasites was measured as described previously [5]. Briefly, L_1_ larvae were recovered from mice (17dpi) by digesting minced diaphragms for 15 min at 37°C in PBS containing 2% FCS and 5mg/ml collagenase I (Sigma). Larvae were treated with 70% ethanol (warm up at 56°C) overnight. Larvae were then centrifuged and resuspended in 5% glycerol/70% ethanol for one day before cytospin. The cytospin slides were fixed with methanol and stained with HEMA-3 (Fisher Healthcare), and measurements were performed using a BX51 microscope fitted with DP-12 digital camera system (Olympus, Melville, N.Y.). The area of each larva was calculated using Microsuite Basic software. At least 25 larvae were measured per mouse, measurements from 3–4 mice per group were pooled, and values expressed in μm^2^.

Note that the cytokines under investigation are known to influence intestinal *Trichinella*. Survival of adult worms in the gut determines the number of NBL released and the dose of NBL arriving in muscle. In order to control for this variability, we used IV infection with NBL in some strains of mice. When measured, larvae collected from IV infections at 13 days are consistently larger than larvae collected from oral infections at 17 dpi, possibly due to the synchronized nature of IV infection. In order to make this distinction more apparent in the results, experiments in which mice were infected IV are graphed on one scale (25,000 μm^2^ max) and data from mice infected orally are graphed on a different scale (15,000 μm^2^ max).

### ELISA

Antigen restimulation of cells from cervical lymph nodes (CLN) of WT and STAT6^-/-^ mice and assay of IL-10 were performed as described previously [16].

### Bone marrow chimeras

6- to 8-week old B6.SJL (CD45.1) or STAT6^-/-^ (CD45.2) were provided with acidified water (pH 2–3) containing 1 mg/ml gentamicin sulfate solution 1 week prior to lethal gamma irradiation (950 cGy), followed by retro-orbital injection with 10^7^ bone marrow cells isolated from femurs of STAT6^-/-^ or B6.SJL mice, respectively. Reconstitution was confirmed by detection of leukocytes bearing congenic markers by flow cytometry eight weeks after transplantation.

### Flow cytometry

Spleen cells in bone marrow chimeras and controls were incubated with Fc block in 2% FBS containing PBS, followed by staining with FITC-conjugated anti-mouse CD45.1 (Biolegend) and Alexa Flour 700-conjugated anti-mouse CD45.2 (eBioscience). The number of CD4^+^IL-10^+^ cells per diaphragm was determined as described previously [9]. Data were acquired using a Gallios flow cytometer (Beckman Coulter) and analyzed with FlowJo software (Tree Star).

### Eosinophil transfer experiments

Eosinophils were recovered from uninfected IL-5Tg^+^, infected IL-5Tg^+^, IL-5Tg^+^/IL-4^-/-^ or IL-5Tg^+^/STAT6^-/-^ mice 12–20 dpi. Cells were pooled from spleens and peritoneal lavage fluid and purified on magnetic beads as previously described [44]. Briefly, eosinophils were labeled with PE-conjugated anti-Siglec-F antibody (BD) and anti-PE microbeads (Miltenyi Biotec). Average purity of eosinophils from this procedure was >93%. After washing twice with PBS, 5 × 10^6^ eosinophils were resuspended in 200 μl sterile PBS and injected IV into recipient mice three times on alternate days, beginning 5 days post oral infection or on the day of IV infection.

### Immunohistochemistry

Immunohistochemistry was conducted as described previously [45]. Rabbit polyclonal anti-GLUT4 antibody (Novus Biological), rabbit monoclonal anti-phospho-Akt (Ser^473^, Cell Signaling) and anti-total Akt (Cell Signaling) antibodies were used. Sections were examined and photographed on a BX51 microscope.

### Transcriptional profiling and functional enrichment analysis

In order to assess gene expression at the initiation of infection by NBL, diaphragms from WT, Rag1^-/-^ and PHIL mice were harvested at 0, 2 and 7 days post injection of NBL and preserved in RNAlater (Qiagen). Whole diaphragms were homogenized using a FastPrep-24 Sample Preparation System (MP Biomedicals) and RNA was isolated using the miRNeasy kit (Qiagen). Biotin-labeled complementary RNA (cRNA) was generated using the Illumina TotalPrep RNA amplification kit (Ambion). Illumina MouseRef-8 v2.0 expression Beadarrays were hybridized with cRNA from three diaphragms collected on 2 and 7 days post injection, or two diaphragms collected from uninfected Rag1^-/-^ mice. Scanned images were converted to raw expression using GenomeStudio v1.8 software (Illumina). Data analysis was carried out using the statistical computing environment, R (v3.0.2). Raw data was background subtracted, variance stabilized and normalized by robust spline normalization using the Lumi package [46]. Differentially expressed genes (≥2-fold, P<0.05) were identified by linear modeling and Bayesian statistics using the Limma package [47]. Clusters of co-regulated genes were identified by Pearson correlation using the hclust function of the stats package in R. Gene set enrichment analysis was conducted using GSEA software from Broad Institute [48]. Data have been deposited on the Gene Expression Omnibus (GEO) database for public access (GSE67136).

### Code availability

We have provided a supplementary code document including all R codes for the analysis (S1 Code).

### Statistical analysis

All experiments were performed twice with similar results. Means ± SD were calculated from data collected from individual mice unless otherwise indicated. Significant differences were determined using Student’s *t* test or One-way ANOVA with Tukey’s post hoc test for multiple means. Statistical analysis was performed with GraphPad Prism 5 software.

## Supporting Information

S1 Table Genes differentially regulated on 0, 2 and 7 days post IV infection in Rag1^-/-^ mice. The fold change of each gene was expressed as the log2 value.
(XLSX)

S2 Table Genes differentially regulated in uninfected and infected WT and PHIL mice on 0, 2 and 7 days post IV infection. The fold change of each gene was expressed as the log2 value.
(XLSX)

S1 Code Microarray analysis revealed the differentially regulated genes in Rag1^-/-^, WT and PHIL mice on 0, 2, and 7 days post IV infection. All R code used to analyze the microarray data is included.
(PDF)
