# Supplementary material for: Eosinophils and IL-4 Support Nematode Growth Coincident with an Innate Response to Tissue Injury
Source: PLoS Pathog. 2015 Dec 31;11(12):e1005347. doi: 10.1371/journal.ppat.1005347 (PMC4697774; doi:10.1371/journal.ppat.1005347)
Supplement: S1 Code — All R code used to analyze the microarray data is included. (PDF) [file ppat.1005347.s003.pdf]

Supplementary Code for manuscript entitled - *Eosinophil-derived IL-4 promotes nematode growth in an innate context*

Lu Huang, Daniel P. Beiting, Nebiat G. Gebreselassie, Lucille F. Gagliardo, Maura C. Ruyechan, Nancy A. Lee, James J. Lee, Judith A. Appleton

September, 2015

Contents

Abstract **2**

R packages **2**

Set-up and QC of data from *Rag1*$^{-/-}$ exper **3**

Figure 1 - housekeeping Genes: 3

Figure 2 - signal distribution before normalization: 3

Figure 3 - signal distribution after normalization 3

Exploratory analysis of *Rag1*$^{-/-}$ data **7**

Figure 4: hierarchical clustering 7

Figure 5 - Principal component analysis (PCA) 8

Figure 6 - PCA 'small multiples' graph: 8

Identification of Differentially Expressed Genes in *Rag1*$^{-/-}$ mice **9**

Table 1 - top 20 differentially expressed genes (DEGs); 2dpi vs naive 10

Table 2 - top 20 DEGs; 7dpi vs naive 10

Table 3 - top 20 DEGs; 7dpi vs 2dpi 11

Figure 7: venn diagram of DEGs (>= 1.5 fold up/down and FDR of <= 0.05) 12

Clustering of differentially expressed genes from *Rag1*$^{-/-}$ exper **13**

Figure 8 - heatmap of DEGs 13

Figure 9 - heatmap of cluster 1 13

Figure 10 - selected genes from cluster 1 15

Figure 11 - heatmap of cluster 2 16

Figure 12 - selected genes from cluster 2 16

Figure 13 - heatmap of cluster 3 18

Figure 14 - selected genes from cluster 3 18

Figure 15 - heatmap of cluster 4 20

Figure 16 - selected genes from glycolytic cycle 22

Set-up and QC of data from *WT vs PHIL* exper 22

Figure 17 - housekeeping Genes . . . 23

Figure 18 - signal distribution before normalization . . . 23

Figure 19 - signal distribution after normalization . . . 24

Exploratory analysis of *WT vs PHIL* exper 26

Figure 20 - hierarchical clustering . . . 26

Figure 21 - Principal component analysis . . . 27

Figure 22 - PCA 'small multiples' graph . . . 27

Identification of Differentially Expressed Genes in *WT vs PHIL* exper 28

Table 4 - top 20 DEGs; WT vs PHIL at 7dpi . . . 29

Clustering of differentially expressed genes from *WT vs PHIL* exper 30

Figure 23 - heatmap for WT/PHIL data . . . 30

Figure 24 - selected genes from comparison of WT vs PHIL mice . . . 30

Session Info 32

Abstract

This document contains all the code used to analyze the microarray data in the manuscript entitled: *Eosinophil-derived IL-4 promotes nematode growth in an innate context*. Before this analysis was carried out, raw image scans of the Illumina BeadArrays were converted to non-normalized, non-background subtracted data using GenomeStudio Software from Illumina (see methods section of manuscript for more details). Raw data is available on the Gene Expression Omnibus (GEO) under accession GSE67136.

R packages

These are the R/bioconductor packages used for this analysis:

```
library(lumi)
library(lumiMouseIDMapping)
library(lumiMouseAll.db)
library(RColorBrewer)
library(gplots)
library(ggplot2)
library(genefilter)
library(limma)
library(annotate)
library(reshape2)
library(dplyr)
library(Biobase)
```

This dynamic html summary report was compiled in Rmarkdown using the following packages:

```
library(rmarkdown)
library(knitr)
```

Set-up and QC of data from *Rag1*$^{-/-}$ exper

I begin by reading in a simple text file that describes the design of the study. I use this file to set treatment groups, sample labels, etc.

```
targets.RAG <- read.delim("Trichinella_studyDesign_exper1.txt", sep="\t")
groups.RAG <- paste(targets.RAG$treatment, targets.RAG$genotype, sep=".")
groups.RAG <- factor(groups.RAG)
#now capture sample names from this file
sampleLabels <- targets.RAG$name
```

Now reading in the raw array data:

```
rawData <- lumiR("FinalReport_probes_samples.txt", convertNuID = TRUE,
                 sep = NULL, detectionTh = 0.01, na.rm = TRUE,
                 lib = "lumiMouseIDMapping")
#subset data to separate _Rag1^-/-^_ data from WT and PHIL data
rawData_WT.PHIL <- rawData[,-1:-8]
rawData_RAG <- rawData[,1:8]
summary(rawData, 'QC')
```

Figure 1 - housekeeping Genes:

As a crude measure of array quality and consistency across arrays, take a look at how a set of housekeeping genes behaved on each of the 9 arrays

```
#Read control probe data into a separate LumiBatch and take a look at these controls
myControlData <- addControlData2lumi("FinalReport_probes_controls.txt", rawData)
myControlData.RAG <- myControlData[,1:8]
#subset to get just the data from RAG mice
plotHousekeepingGene(myControlData.RAG, addLegend=F)
```

Figure 2 - signal distribution before normalization:

Now we'll look at the distribution of signal intensity from each array (note: this is before any normalization or filtering is applied to the data)

```
#choose a color scheme for the next graph
cols <- topo.colors (n=8, alpha=1)
hist(rawData_RAG, xlab = "log2 expression", main = "non-normalized data - histograms", col=cols)
```

Figure 3 - signal distribution after normalization

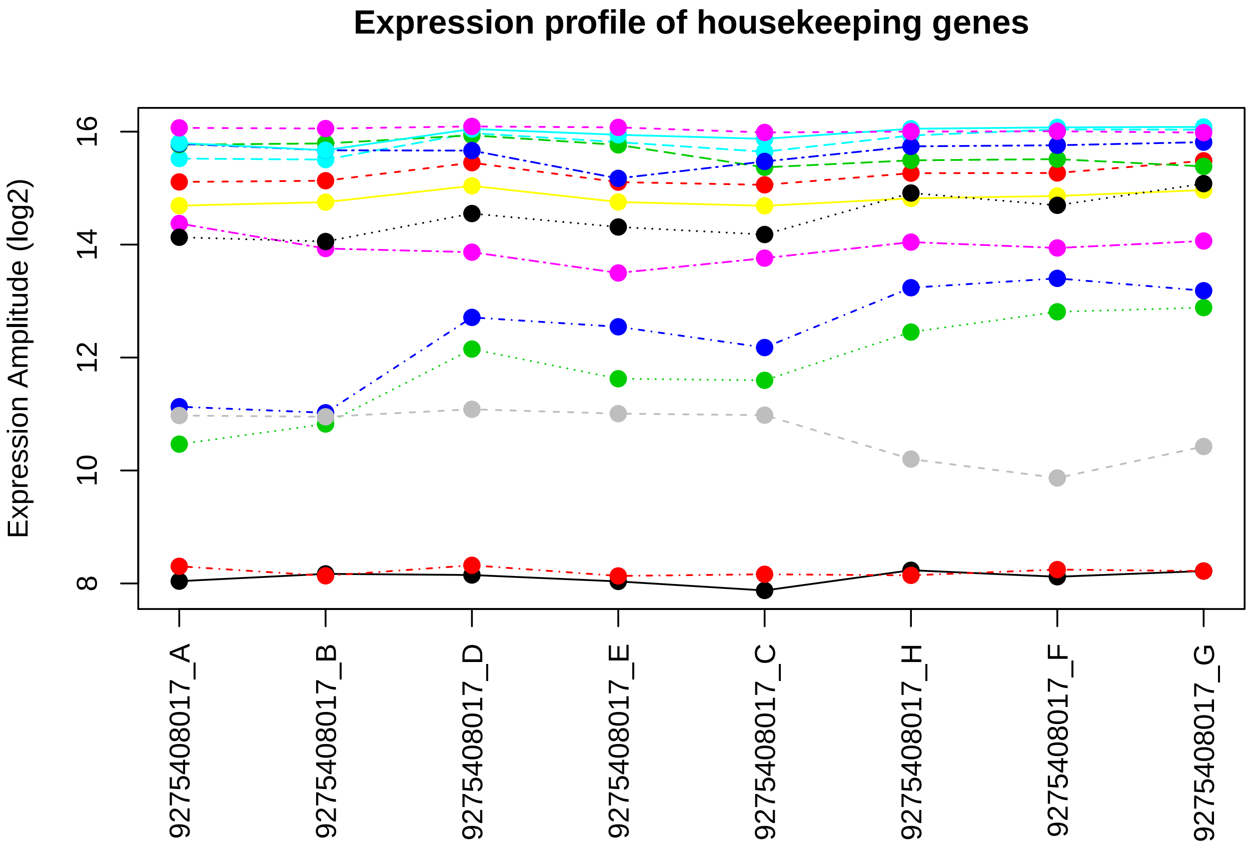

Figure 1: housekeeping genes from *Rag1*$^{-/-}$ arrays

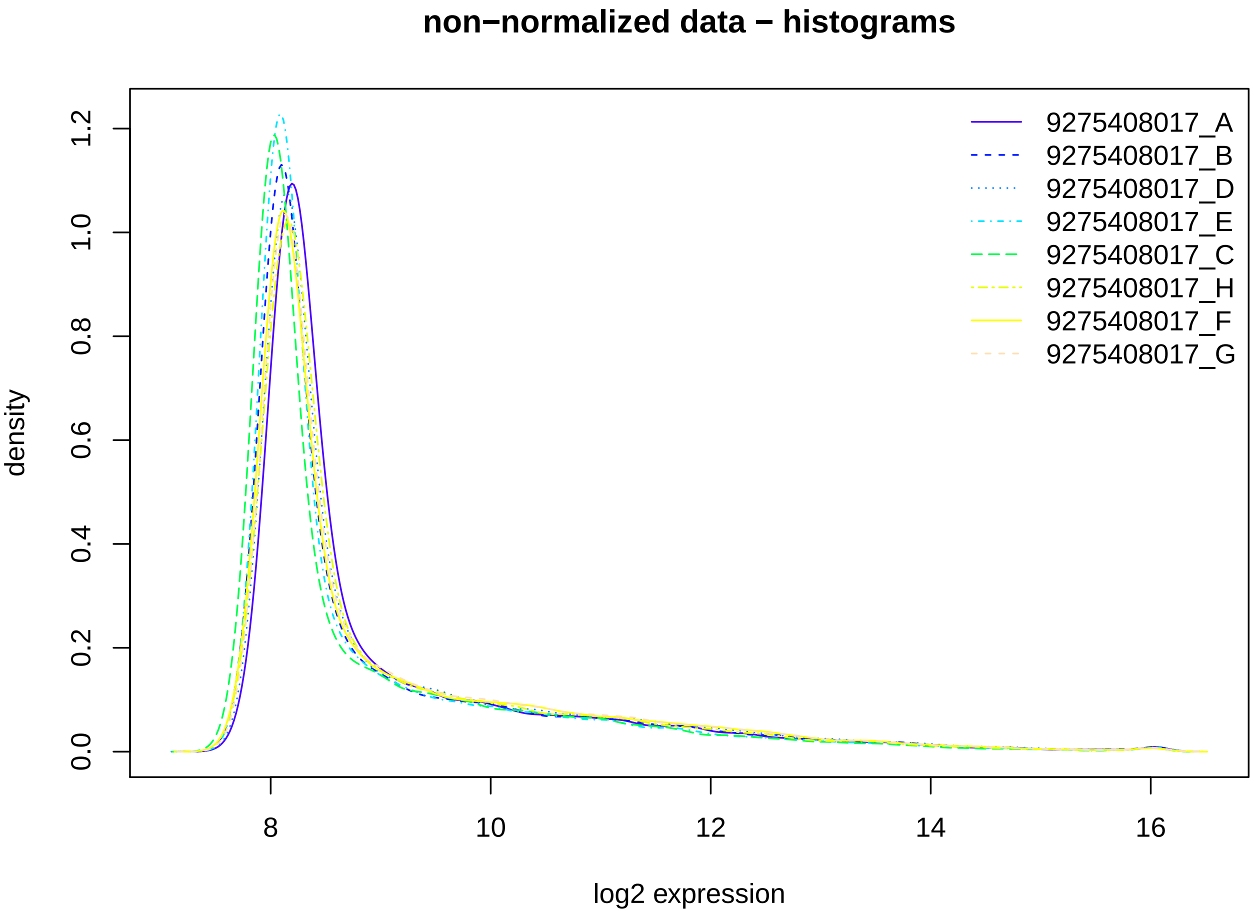

Figure 2: signal distribution from *Rag1*-/- arrays before normalization

```
hist(normData, xlab = "log2 expression", main = "normalized data - histograms", col=cols)
```

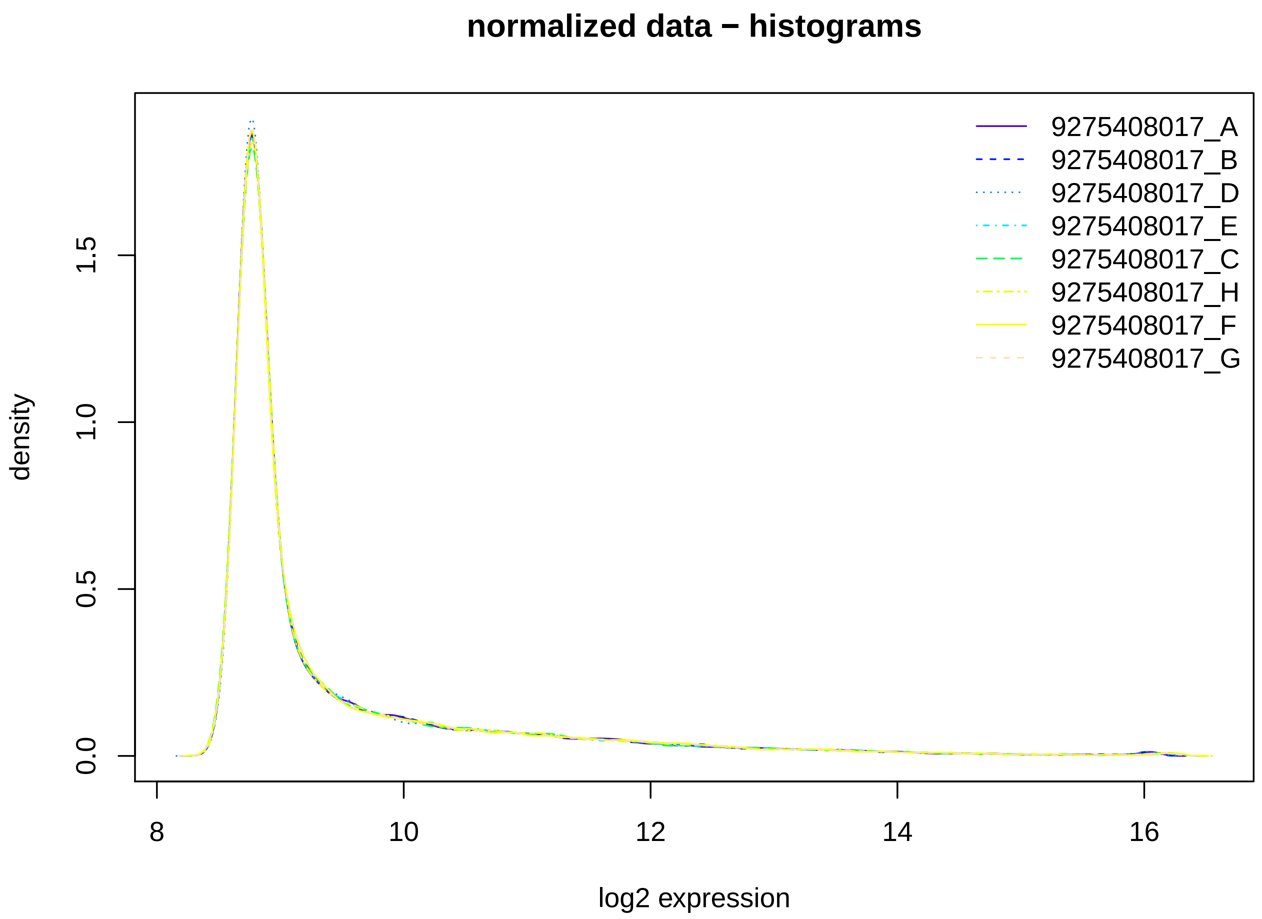

Figure 3: signal distribution from *Rag1*$^{-/-}$ arrays after robust spline normalization

Filtering data: Removing probes that were not detected above background, probes that had low variation, and genes without an EntrezID

```
filtered_geneList <- nsFilter(normData, require.entrez=TRUE,
                              remove.dupEntrez=TRUE, var.func=IQR,
                              var.filter=TRUE, var.cutoff=0.5, filterByQuantile=TRUE)
# extract the ExpressionSet from this filtered list.
filtered.eset <- filtered_geneList$eset
#now convert to a datamatrix that will contain only the probes after filtering
filtered.matrix <- as.matrix(filtered.eset)
probeList <- rownames(filtered.matrix)
```

Exploratory analysis of *Rag1*$^{-/-}$ data

Figure 4: hierarchical clustering

```
distance <- dist(t(filtered.matrix),method="maximum")
clusters <- hclust(distance, method = "complete")
plot(clusters, label = sampleLabels, main="Trichinella in Rag1 KO mice")
```

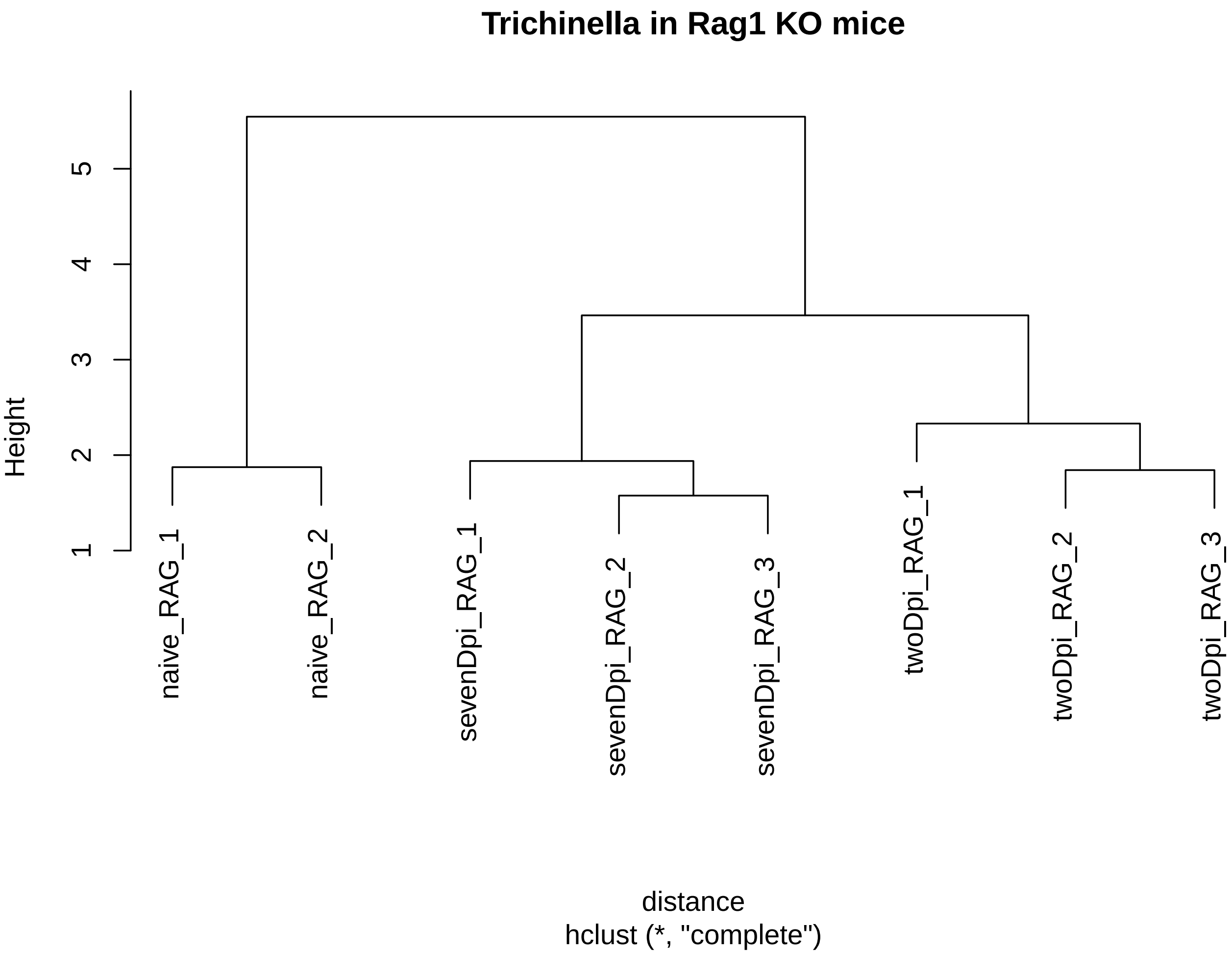

Figure 4: hierarchical clustering dendogram of *Rag1*$^{-/-}$ data

PCA: carrying out a Principle Component Analysis. Result shows that first two principle components account for 80% of the variation in the data

```
pca.res <- prcomp(t(filtered.matrix), scale.=F, retx=T)
ls(pca.res)
summary(pca.res)
head(pca.res$rotation)
head(pca.res$x)
#plot(pca.res, las=1)
pc.var<-pca.res$sdev^2
```

```
pc.per<-round(pc.var/sum(pc.var)*100, 1)
pc.per
```

Figure 5 - Principal component analysis (PCA)

```
data.frame <- as.data.frame(pca.res$x)
ggplot(data.frame, aes(x=PC1, y=PC2, colour=factor(groups.RAG))) +
  geom_point(size=5) +
  theme(legend.position="right")
```

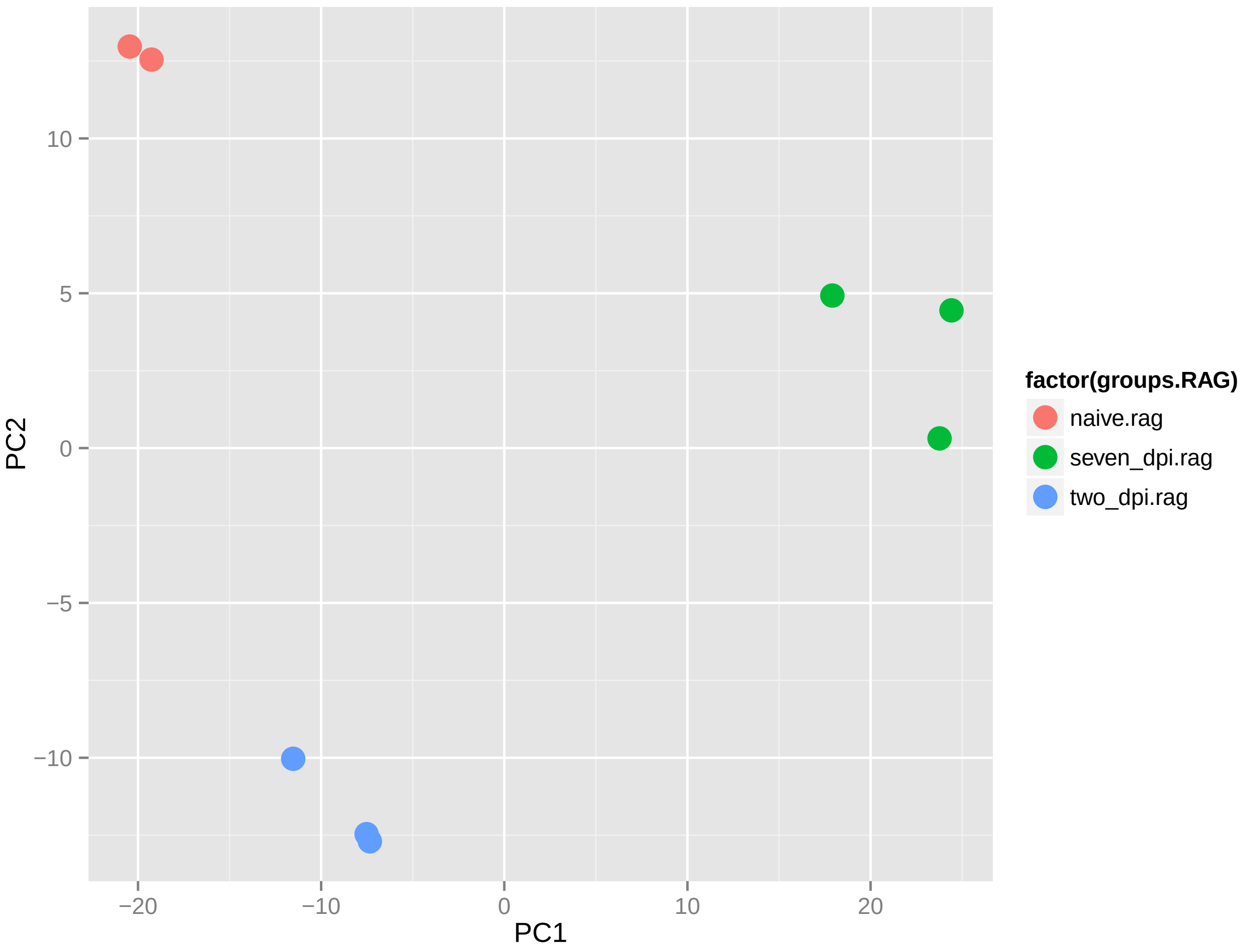

Figure 5: PCA plot of *Rag1*$^{-/-}$ data

Figure 6 - PCA 'small multiples' graph:

```
melted <- cbind(groups.RAG, melt(pca.res$x[,1:4]))
#look at your 'melted' data
ggplot(melted) +
```

```
  geom_bar(aes(x=Var1, y=value, fill=groups.RAG), stat="identity") +
  facet_wrap(~Var2)
```

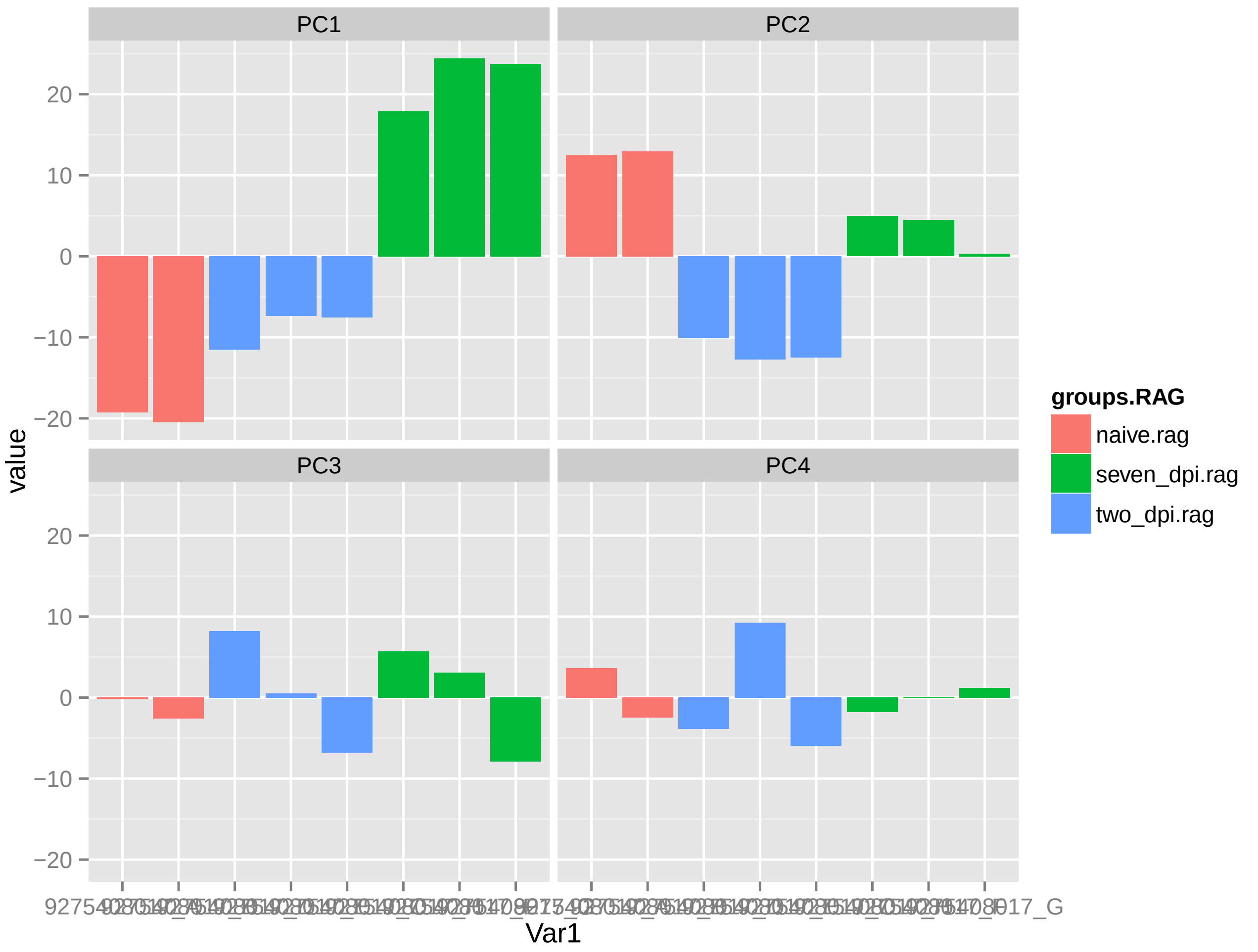

Figure 6: PCA 'small multiples' plot of *Rag1*$^{-/-}$ data

Setting up experimental design: This is a critical point in our analysis where we can begin to ask specific questions about which, if any, genes were differentially expressed. To do this, we must first define our questions in the form of a model matrix

```
design.RAG <- model.matrix(~0+groups.RAG)
colnames(design.RAG) <- levels(groups.RAG)
design.RAG
```

Identification of Differentially Expressed Genes in *Rag1*$^{-/-}$ mice

Fitting linear model to data and set-up contrast matrix

```
fit.RAG <- lmFit(filtered.matrix, design.RAG)
#add annotation into the linear model
fit.RAG$genes$Symbol <- getSYMBOL(probeList, "lumiMouseAll.db")
```

```
fit.RAG$genes$Entrez <- getEG(probeList, "lumiMouseAll.db")
# set up a contrast matrix based on the pairwise comparisons of interest
contrast.matrix.RAG <- makeContrasts(early_RAG = two_dpi.rag - naive.rag,
                                     late_RAG = seven_dpi.rag - naive.rag,
                                     late_vs_early = seven_dpi.rag - two_dpi.rag,
                                     levels=design.RAG)
fits.RAG <- contrasts.fit(fit.RAG, contrast.matrix.RAG)
ebFit.RAG <- eBayes(fits.RAG)
```

Table 1 - top 20 differentially expressed genes (DEGs); 2dpi vs naive

Given that our hierarchical cluster dendogram of all the samples showed pretty good separation of the three treatment groups, let's start by just asking to see the top 20 genes most significantly different between 2dpi vs naive *Rag1*$^{-/-}$ mice.

```
# use topTable function to take a look at the top most differentially expressed genes between
probeset.list <- topTable(ebFit.RAG, adjust ="BH", coef=1, number=20, sort.by="logFC")
row.names(probeset.list) <- probeset.list[,1]
probeset.list <- probeset.list[,c(2,3,7)]
knitr::kable(probeset.list, caption="top 20 differentially expressed genes (DEGs); 2dpi vs naive")
```

Table 1: top 20 differentially expressed genes (DEGs); 2dpi vs naive

	ID.Entrez	logFC	adj.P.Val
Myl4	17896	4.976799	0.0000016
Igtp	16145	4.180690	0.0000152
Chrng	11449	3.422225	0.0000170
Ankrd1	107765	3.417938	0.0000218
Irgm2	54396	3.399053	0.0000153
Lgals3	16854	3.208877	0.0000092
Fcgr4	246256	3.203660	0.0000359
Gbp2	14469	3.046538	0.0001752
Aif1l	108897	2.987109	0.0000139
Cxcl9	17329	2.973632	0.0000039
Irgm1	15944	2.572033	0.0000983
Mustn1	66175	2.516210	0.0000005
Gbp3	55932	2.456989	0.0002454
Psmb10	19171	2.434089	0.0000271
Hist1h2ap	319171	2.410692	0.0000271
Cd274	60533	2.344000	0.0000983
Vmn1r65	81013	2.234895	0.0002454
Myh8	17885	2.210298	0.0011715
H2-Eb1	14969	2.103345	0.0000572
Cdkn1a	12575	2.082754	0.0000351

Table 2 - top 20 DEGs; 7dpi vs naive

```
# use topTable function to take a look at the top most differentially expressed genes between
probeset.list <- topTable(ebFit.RAG, adjust ="BH", coef=2, number=20, sort.by="logFC")
```

```
row.names(probeset.list) <- probeset.list[,1]
probeset.list <- probeset.list[,c(2,3,7)]
knitr::kable(probeset.list, caption="top 20 DEGs; 7dpi vs naive")
```

Table 2: top 20 DEGs; 7dpi vs naive

	ID.Entrez	logFC	adj.P.Val
Myl4	17896	3.984965	1.40e-06
Tnc	21923	3.403456	3.00e-07
Fcgr4	246256	3.319438	6.20e-06
Myh8	17885	3.278597	2.05e-05
Lox	16948	3.215422	3.00e-07
Hist1h2ap	319171	3.170201	1.40e-06
H2-Ab1	14961	2.929314	5.10e-06
H2-Eb1	14969	2.919959	2.00e-06
Cotl1	72042	2.766649	2.00e-06
Wisp2	22403	2.760086	2.00e-07
Nt5e	23959	2.745927	3.00e-07
Cd74	16149	2.680316	3.60e-06
Lgals3	16854	2.641140	5.20e-06
Sqle	20775	2.611099	3.00e-07
Actg2	11468	2.609393	3.70e-06
C1qb	12260	2.594460	4.00e-06
Ctgf	14219	2.573980	8.00e-07
Hist2h2ac	319176	2.484988	5.70e-06
Fcer1g	14127	2.399575	1.25e-05
Cxcl9	17329	2.385419	3.70e-06

Table 3 - top 20 DEGs; 7dpi vs 2dpi

```
# use topTable function to take a look at the top most differentially expressed genes between
probeset.list <- topTable(ebFit.RAG, adjust ="BH", coef=3, number=20, sort.by="logFC")
row.names(probeset.list) <- probeset.list[,1]
probeset.list <- probeset.list[,c(2,3,7)]
knitr::kable(probeset.list, caption="top 20 DEGs; 7dpi vs 2dpi")
```

Table 3: top 20 DEGs; 7dpi vs 2dpi

	ID.Entrez	logFC	adj.P.Val
Chrng	11449	-3.055479	0.0000036
Actg2	11468	2.899613	0.0000011
Lox	16948	2.849436	0.0000003
Tnc	21923	2.820338	0.0000003
Nt5e	23959	2.784003	0.0000001
Wisp2	22403	2.743014	0.0000001
Dkk3	50781	2.366427	0.0000002
Sqle	20775	2.283830	0.0000003
Acta2	11475	2.266771	0.0000099
Chst8	68947	2.216890	0.0000001

	ID.Entrez	logFC	adj.P.Val
Slc24a3	94249	2.216242	0.0000013
Nupr1	56312	2.178814	0.0000019
Ctgf	14219	2.178057	0.0000011
Csrp3	13009	-2.098073	0.0001586
Hp	15439	2.094226	0.0010049
Cacna1h	58226	2.093328	0.0000106
Igfbp2	16008	2.064437	0.0000003
Hmgn3	94353	2.053752	0.0000020
Igtp	16145	-2.017003	0.0001073
Cd274	60533	-1.960577	0.0000305

Figure 7: venn diagram of DEGs (>= 1.5 fold up/down and FDR of <= 0.05)

```
# use the 'decideTests' function to 'test' for differential expression
results <- decideTests(ebFit.RAG, method="global", adjust.method="BH", p.value=0.05, lfc=0.59)
vennDiagram(results, include="both", fig.cap="Venn diagram of DEGs from _Rag1^-/-^_ exper")
```

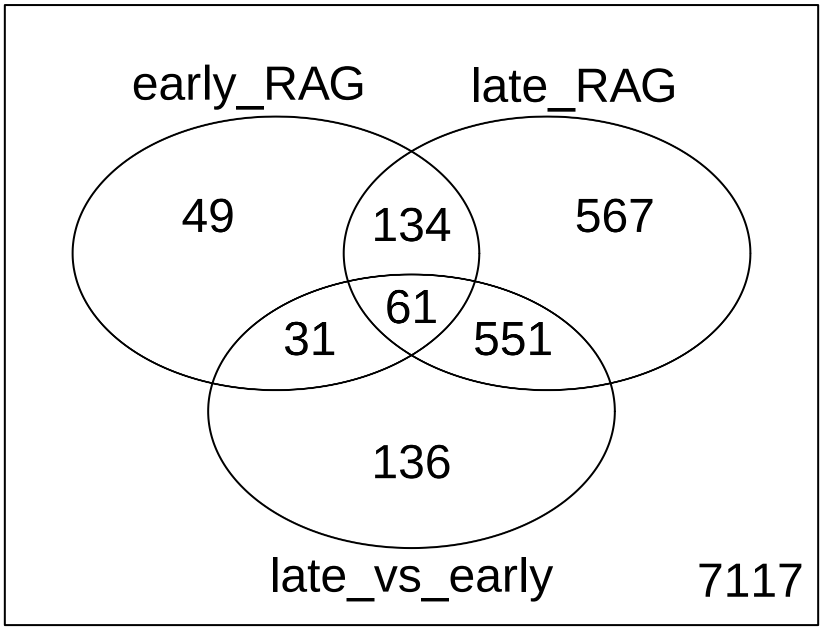

Figure 7: Venn diagram of DEGs from *Rag1*$^{-/-}$ exper

pull out these differentially expressed genes

```
diffProbes <- which(results[,1] !=0 | results[,2] !=0 | results[,3] !=0)
diffSymbols <- fit.RAG$genes$Symbol[results[,1] !=0 | results[,2] !=0 | results[,3] !=0]
diffEntrez <- fit.RAG$genes$Entrez[results[,1] !=0 | results[,2] !=0 | results[,3] !=0]
#convert to an expressionSet object
myEset <- new("ExpressionSet", exprs = filtered.matrix)
#link the eset to annotation data
annotation(myEset) <- "lumiMouseAll.db"
diffData <- myEset[results[,1] !=0 | results[,2] !=0 | results[,3] !=0]
```

```
#pull the expression data back out of the eset object
diffData <- exprs(diffData)
dim(diffData)
write.table(cbind(diffSymbols, diffEntrez, diffData),
            "diffGenes_RAG.xls", sep="\t", quote=FALSE)
```

average biological replicates so we can make the least cluttered heatmap possible

```
head(diffData)
colnames(diffData) <- groups.RAG
rownames(diffData) <- diffSymbols
head(diffData)
diffData.AVG <- avearrays(diffData)
head(diffData.AVG)
```

Clustering of differentially expressed genes from *Rag1$^{-/-}$* exper

Figure 8 - heatmap of DEGs

make heatmap from the 1529 differentially expressed genes that were identified above. *This heatmap appears in Figure 5a of the manuscript*

```
#cluster rows by pearson correlation
hr <- hclust(as.dist(1-cor(t(diffData.AVG), method="pearson")), method="average")
#cluster columns by spearman correlation
hc <- hclust(as.dist(1-cor(diffData.AVG, method="spearman")), method="complete")
# Cut the resulting tree and create color vector for clusters.
mycl <- cutree(hr, k=6)
mycolhc <- rainbow(length(unique(mycl)), start=0.1, end=0.9)
mycolhc <- mycolhc[as.vector(mycl)]
myheatcol <- greenred(75)
#plot the hclust results as a heatmap
heatmap.2(diffData.AVG, Rowv=as.dendrogram(hr),
          Colv=NA, col=myheatcol, scale="row", labRow=NA,
          density.info="none", trace="none", RowSideColors=mycolhc,
          cexRow=1.5, cexCol=1, key=T, keysize=1, margins=c(10,30))
```

Figure 9 - heatmap of cluster 1

based on the heatmap above at least 4 main clusters stand out as potentially interesting. In the next few code chunks I pull out these four clusters to examine them in more detail. In this document I will refer to the clusters by the numbering shown in the manscript. The first cluster includes genes successively downregulated following infection.

```
clid <- c(2)
ysub <- diffData.AVG[names(mycl[mycl%in%clid]),]
hrsub <- hclust(as.dist(1-cor(t(ysub), method="pearson")), method="complete")
heatmap.2(ysub, Rowv=as.dendrogram(hrsub),
          Colv=NA, col=myheatcol, scale="row",
```

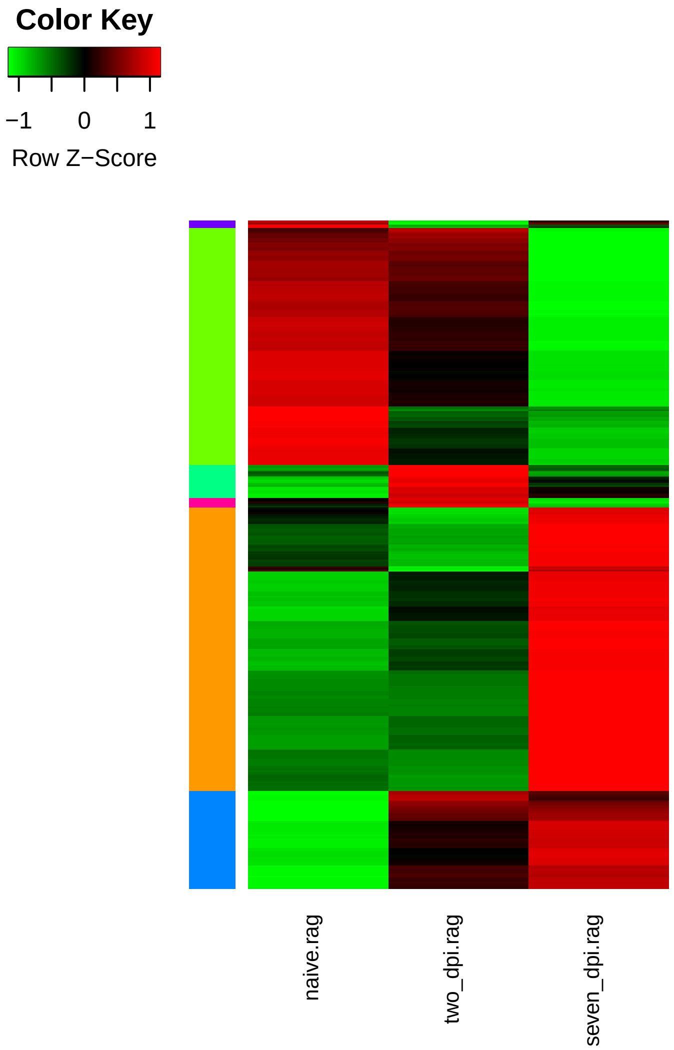

Figure 8: heatmap of *Rag1*$^{-/-}$ DEGs. *Corresponds to Figure 5a in manuscript*

```
          labRow=NA, labCol = NA,
          density.info="none", trace="none",
          RowSideColors=mycolhc[mycl%in%clid],
          key=T, keysize=1, margins=c(10,35))
```

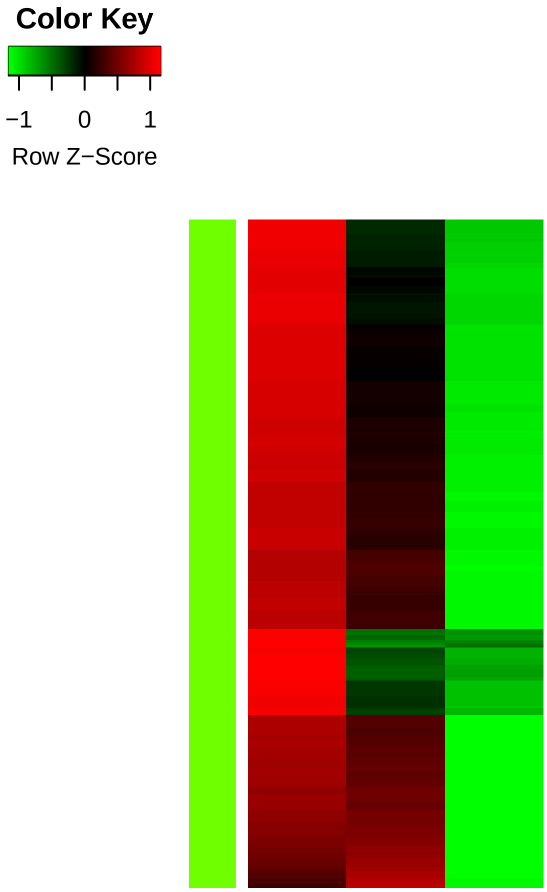

Figure 9: cluster 1 from main heatmap showing 542 genes downregulated during infection. Gene Ontology enrichment analysis of the genes from this cluster showed enrichment for genes involved in muscle function and acetyl-CoA metabolism (corresponds to part of Figure 5b in manuscript)

Figure 10 - selected genes from cluster 1

Six genes were selected from a total of 542 genes in cluster 1. *this plot was used to make part of Figure 5d in the manuscript*

```
clid <- c(2)
#print out row labels in same order as shown in the heatmap
cluster <- data.frame(Labels=rev(hrsub$labels[hrsub$order]))
cluster.symbols <- as.character(cluster[,1])
diffData.frame <- as.data.frame(diffData.AVG)
rows.to.keep <- diffData.frame[cluster.symbols,]
write.table(rows.to.keep,"Cluster2_downreg.xls", sep="\t", quote=FALSE)
mySelected <- as.matrix(read.delim("Cluster2_downreg_selected.txt",
                                   sep="\t", stringsAsFactors = FALSE,
```

```
                              header=TRUE, row.names=1))
heatmap.2(mySelected,
          Rowv=NA, Colv=NA,
          col=myheatcol, scale="row",
          density.info="none", trace="none",
          labCol=NA, cexRow=1.5, cexCol=1,
          key=T, keysize=1, margins=c(20,35))
```

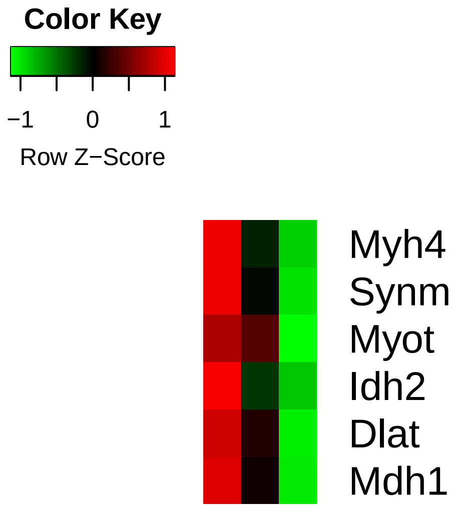

Figure 10: six genes selected from cluster 1 (corresponds to part of Figure 5d in manuscript)

Figure 11 - heatmap of cluster 2

A second prominent cluster of genes in the main heatmap (figure 8, above) includes genes markedly upregulated at 2 dpi, but returning to lower expression by 7 dpi

```
clid <- c(3)
ysub <- diffData.AVG[names(mycl[mycl%in%clid]),]
hrsub <- hclust(as.dist(1-cor(t(ysub), method="pearson")), method="complete")
heatmap.2(ysub, Rowv=as.dendrogram(hrsub),
          Colv=NA, col=myheatcol, scale="row",
          labRow=NA, labCol=NA,
          density.info="none", trace="none",
          RowSideColors=mycolhc[mycl%in%clid],
          key=T, keysize=1, margins=c(10,35))
```

Figure 12 - selected genes from cluster 2

Six genes were selected from a total of 76 genes in cluster 2. *This plot was used to make part of Figure 5d in the manuscript*

```
#print out row labels in same order as shown in the heatmap
cluster <- data.frame(Labels=rev(hrsub$labels[hrsub$order]))
cluster.symbols <- as.character(cluster[,1])
```

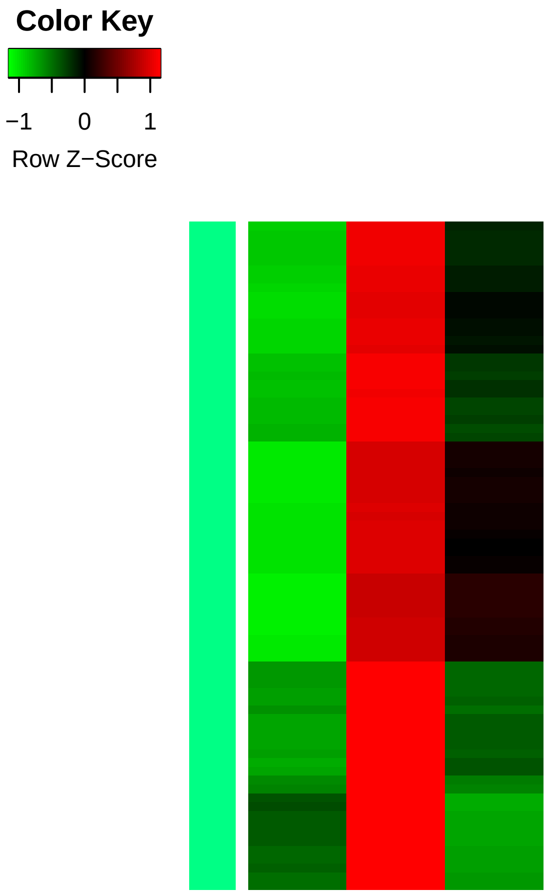

Figure 11: cluster 2 from main heatmap showing 76 genes transiently upregulated during infection. Gene Ontology enrichment analysis of the genes from this cluster showed a slight enrichment of genes involved in host defense (Figure 5b in manuscript). Closer inspection of these genes identified STAT1 and numerous canonical STAT1 targets (Figure 5d in manuscript)

```
diffData.frame <- as.data.frame(diffData.AVG)
rows.to.keep <- diffData.frame[cluster.symbols,]
write.table(rows.to.keep,"Cluster3_inducedEarly.xls", sep="\t", quote=FALSE)
mySelected <- as.matrix(read.delim("Cluster3_STAT1targets_selected.txt",
                               sep="\t", stringsAsFactors = FALSE,
                               header=TRUE, row.names=1))
heatmap.2(mySelected,
          Rowv=NA, Colv=NA,
          col=myheatcol, scale="row",
          density.info="none", trace="none",
          labCol=NA, cexRow=1.5, cexCol=1,
          key=T, keysize=1, margins=c(20,35))
```

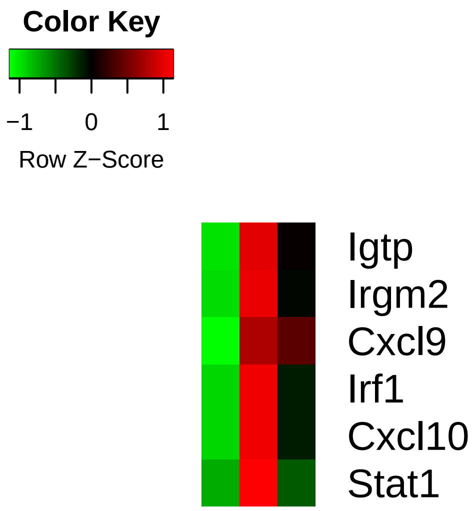

Figure 12: six genes selected from cluster 2 (corresponds to part of Figure 5d in manuscript)

Figure 13 - heatmap of cluster 3

A third prominent cluster of genes in the main heatmap includes genes markedly upregulated only at 7dpi

```
clid <- c(1)
ysub <- diffData.AVG[names(mycl[mycl%in%clid]),]
hrsub <- hclust(as.dist(1-cor(t(ysub), method="pearson")), method="complete")
heatmap.2(ysub, Rowv=as.dendrogram(hrsub),
          Colv=NA, col=myheatcol, scale="row",
          labRow=NA, labCol=NA,
          density.info="none", trace="none",
          RowSideColors=mycolhc[mycl%in%clid],
          key=T, keysize=1, margins=c(10,35))
```

Figure 14 - selected genes from cluster 3

Six genes were selected from a total of 649 genes in cluster 3. *This plot was used to make part of Figure 5d in the manuscript*

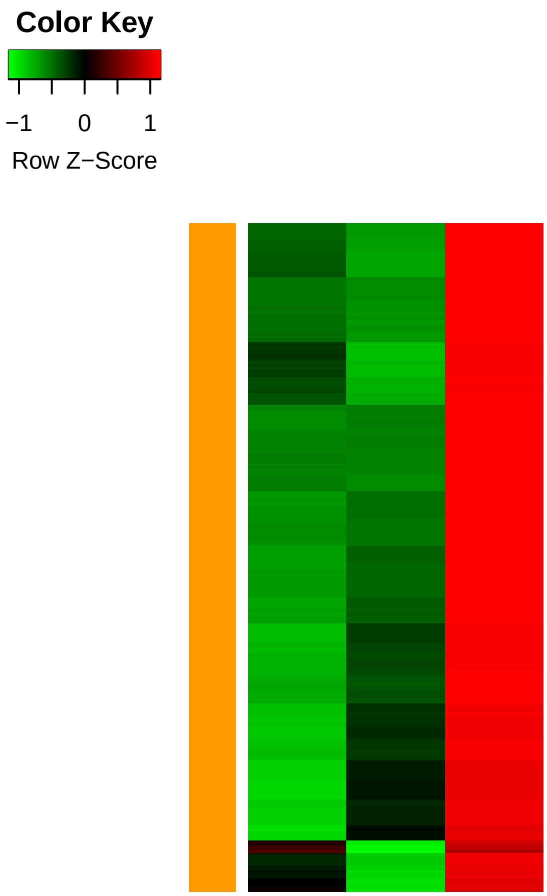

Figure 13: cluster 3 from main heatmap showing 649 genes upregulated late during infection. Gene Ontology enrichment analysis of the genes from this cluster showed an enrichment of genes involved tissue remodeling and repair (Figure 5b in manuscript)

```
#print out row labels in same order as shown in the heatmap
cluster <- data.frame(Labels=rev(hrsub$labels[hrsub$order]))
cluster.symbols <- as.character(cluster[,1])
diffData.frame <- as.data.frame(diffData.AVG)
rows.to.keep <- diffData.frame[cluster.symbols,]
write.table(rows.to.keep,"Cluster1_upreg.xls", sep="\t", quote=FALSE)
mySelected <- as.matrix(read.delim("Cluster1_upreg_selected.txt",
                                   sep="\t", stringsAsFactors = FALSE,
                                   header=TRUE, row.names=1))
heatmap.2(mySelected,
          Rowv=NA, Colv=NA,
          col=myheatcol, scale="row",
          density.info="none", trace="none",
          labCol=NA, cexRow=1.5, cexCol=1,
          key=T, keysize=1, margins=c(20,35))
```

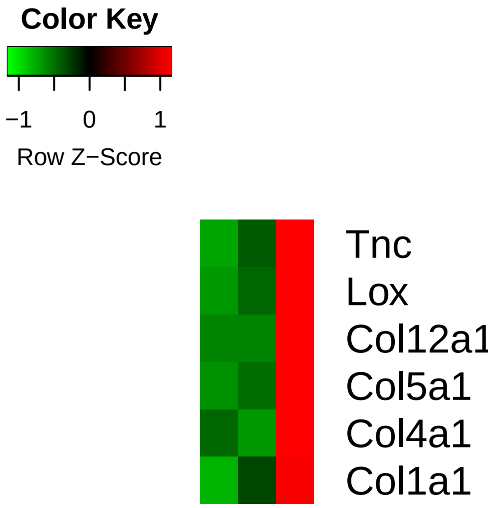

Figure 14: six genes selected from cluster 3 (corresponds to part of Figure 5d in manuscript)

Figure 15 - heatmap of cluster 4

A fourth prominent cluster of genes in the main heatmap were also upregulated strongly by day 7, but they were beginning to rise in expression even at 2 dpi.

```
clid <- c(4)
ysub <- diffData.AVG[names(mycl[mycl%in%clid]),]
hrsub <- hclust(as.dist(1-cor(t(ysub), method="pearson")), method="complete")
heatmap.2(ysub, Rowv=as.dendrogram(hrsub),
          Colv=NA, col=myheatcol, scale="row",
          labRow=NA, labCol=NA,
          density.info="none", trace="none",
          RowSideColors=mycolhc[mycl%in%clid],
          key=T, keysize=1, margins=c(10,35))
```

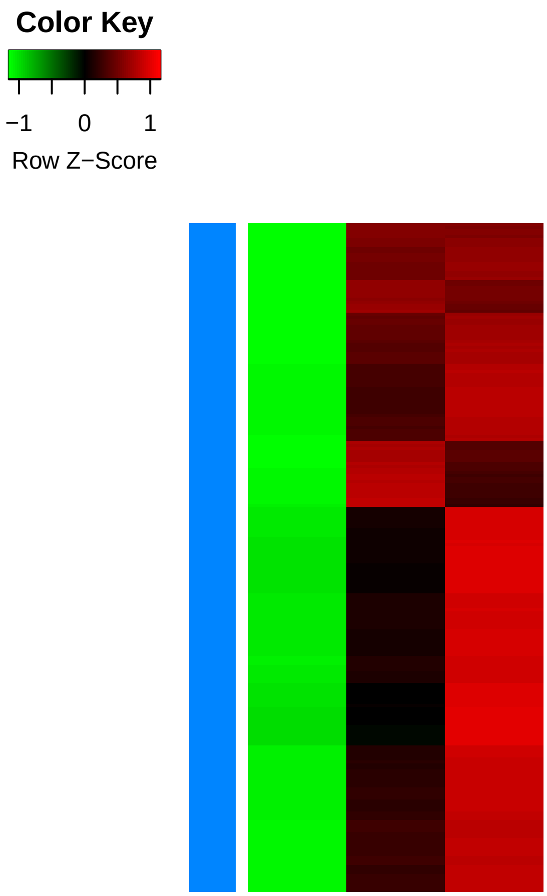

Figure 15: cluster 4 from main heatmap showing 224 genes that begin to be induced by 2dpi and are maintained or further induced at 7dpi. Gene Ontology enrichment analysis of the genes from this cluster showed an enrichment of genes involved antigen presentation and immunity (Figure 5b in manuscript)

```
cluster <- data.frame(Labels=rev(hrsub$labels[hrsub$order]))
cluster.symbols <- as.character(cluster[,1])
diffData.frame <- as.data.frame(diffData.AVG)
rows.to.keep <- diffData.frame[cluster.symbols,]
write.table(rows.to.keep,"Cluster4_upreg.xls", sep="\t", quote=FALSE)
mySelected <- as.matrix(read.delim("Cluster1_upreg_selected.txt",
                               sep="\t", stringsAsFactors = FALSE,
                              header=TRUE, row.names=1))
```

Figure 16 - selected genes from glycolytic cycle

GSEA analysis of 7dpi Rag-/- vs naive shows that glycolytic genes were significantly enhanced. *Six genes were selected from this signature and used to make part of Figure 7c in the manuscript*

```
#print out row labels in same order as shown in the heatmap
mySelected_glycolysis <- read.delim("glycolysis_selected.txt", sep="\t", stringsAsFactors = FALSE, heade
mySelected_glycolysis.matrix <- as.matrix(mySelected_glycolysis)
heatmap.2(mySelected_glycolysis.matrix,
          Rowv=NA, Colv=NA,
          col=myheatcol, scale="row",
          density.info="none", trace="none",
          labCol=NA, cexRow=1.5, cexCol=1,
          key=T, keysize=1, margins=c(20,35))
```

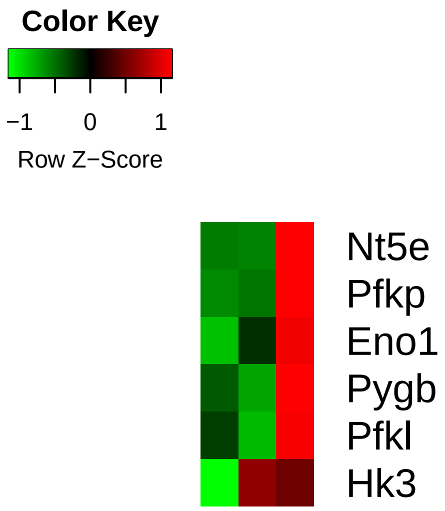

Figure 16: six genes selected from the glycolysis pathway shown to be enriched by GSEA (corresponds to part of Figure 5d in manuscript)

Set-up and QC of data from *WT vs PHIL* exper

define groups and samples

```
targets.PHIL <- read.delim("Trichinella_studyDesign_exper2.txt", sep="\t")
groups.PHIL <- paste(targets.PHIL$treatment, targets.PHIL$genotype, sep=".")
groups.PHIL <- factor(groups.PHIL)
#now capture sample names from this file
sampleLabels.PHIL <- targets.PHIL$name
```

Figure 17 - housekeeping Genes

As a crude measure of array quality and consistency across arrays, take a look at how a set of housekeeping genes behaved on each of the 9 arrays

```
#Read control probe data into a separate LumiBatch and take a look at these controls
myControlData <- addControlData2lumi("FinalReport_probes_controls.txt", rawData)
myControlData_WT.PHIL <- myControlData[,9:24]
#subset to get just the data from RAG mice
plotHousekeepingGene(myControlData_WT.PHIL, addLegend=F)
```

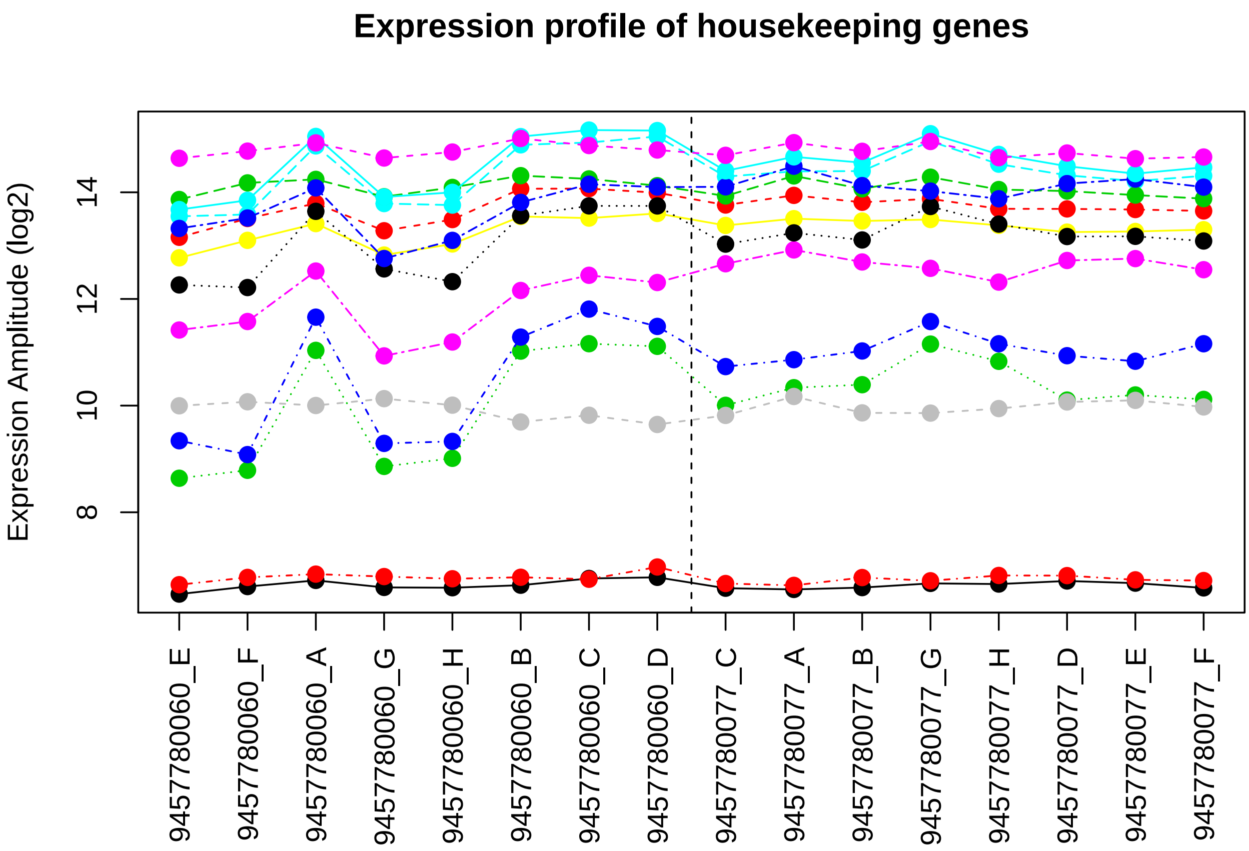

Figure 17: housekeeping genes for WT/PHIL arrays

Figure 18 - signal distribution before normalization

Now we'll look at the distribution of signal intensity from each array (note: this is before any normalization or filtering is applied to the data)

```
#choose a color scheme for the next graph
cols <- topo.colors (n=16, alpha=1)
hist(rawData_WT.PHIL, xlab = "log2 expression", main = "non-normalized data - histograms", col=cols)
```

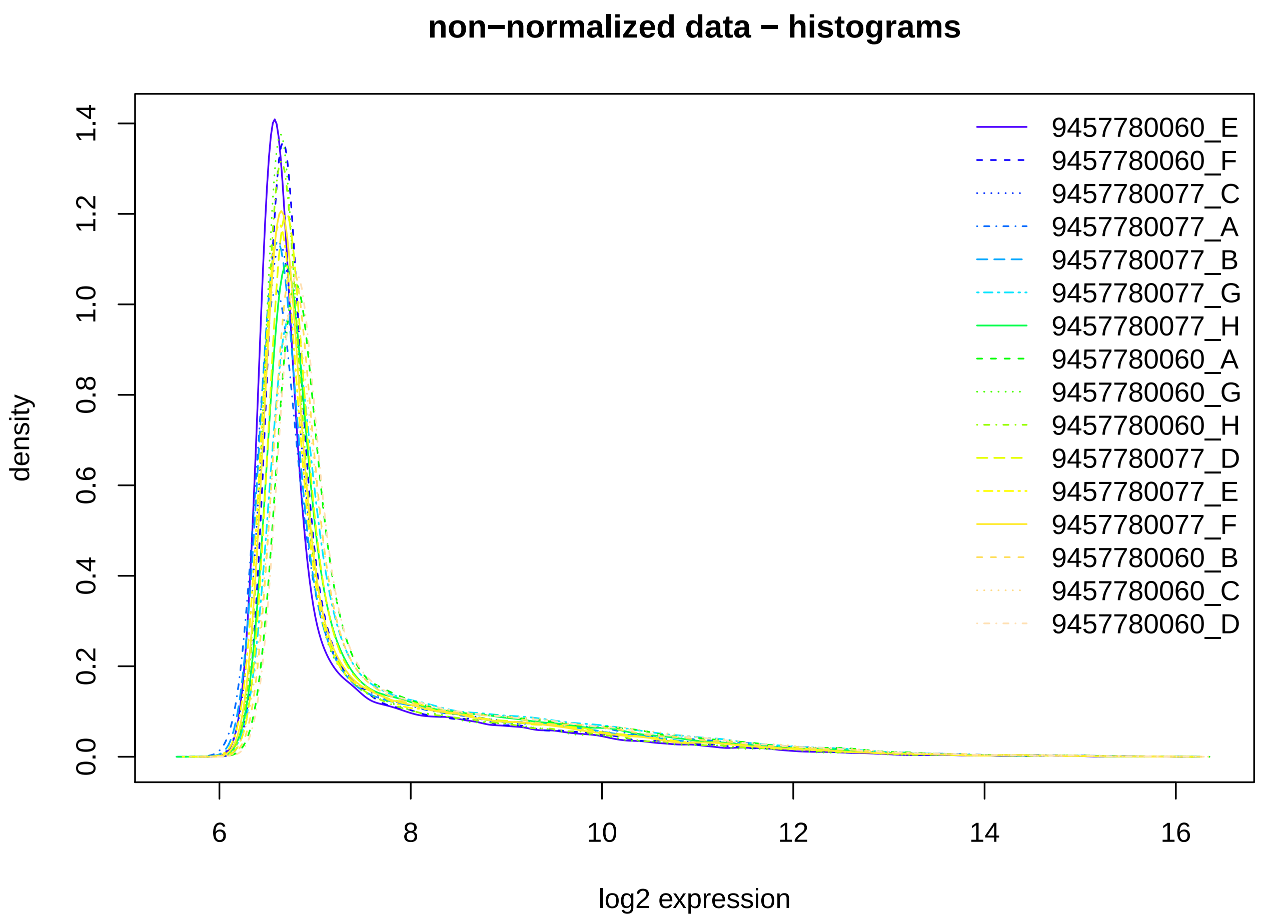

Figure 18: signal distribution for WT/PHIL arrays before normalization

Figure 19 - signal distribution after normalization

```
hist(normData_WT.PHIL, xlab = "log2 expression", main = "normalized data - histograms", col=cols)
```

Filtering data: Removing probes that were not detected above background, probes that had low variation, and genes without an EntrezID

```
filtered_geneList <- nsFilter(normData_WT.PHIL, require.entrez=TRUE,
                              remove.dupEntrez=TRUE, var.func=IQR,
                              var.filter=TRUE, var.cutoff=0.5, filterByQuantile=TRUE)
# extract the ExpressionSet from this filtered list.
filtered.eset <- filtered_geneList$eset
#now convert to a datamatrix that will contain only the probes after filtering
filtered.matrix <- as.matrix(filtered.eset)
probeList <- rownames(filtered.matrix)
```

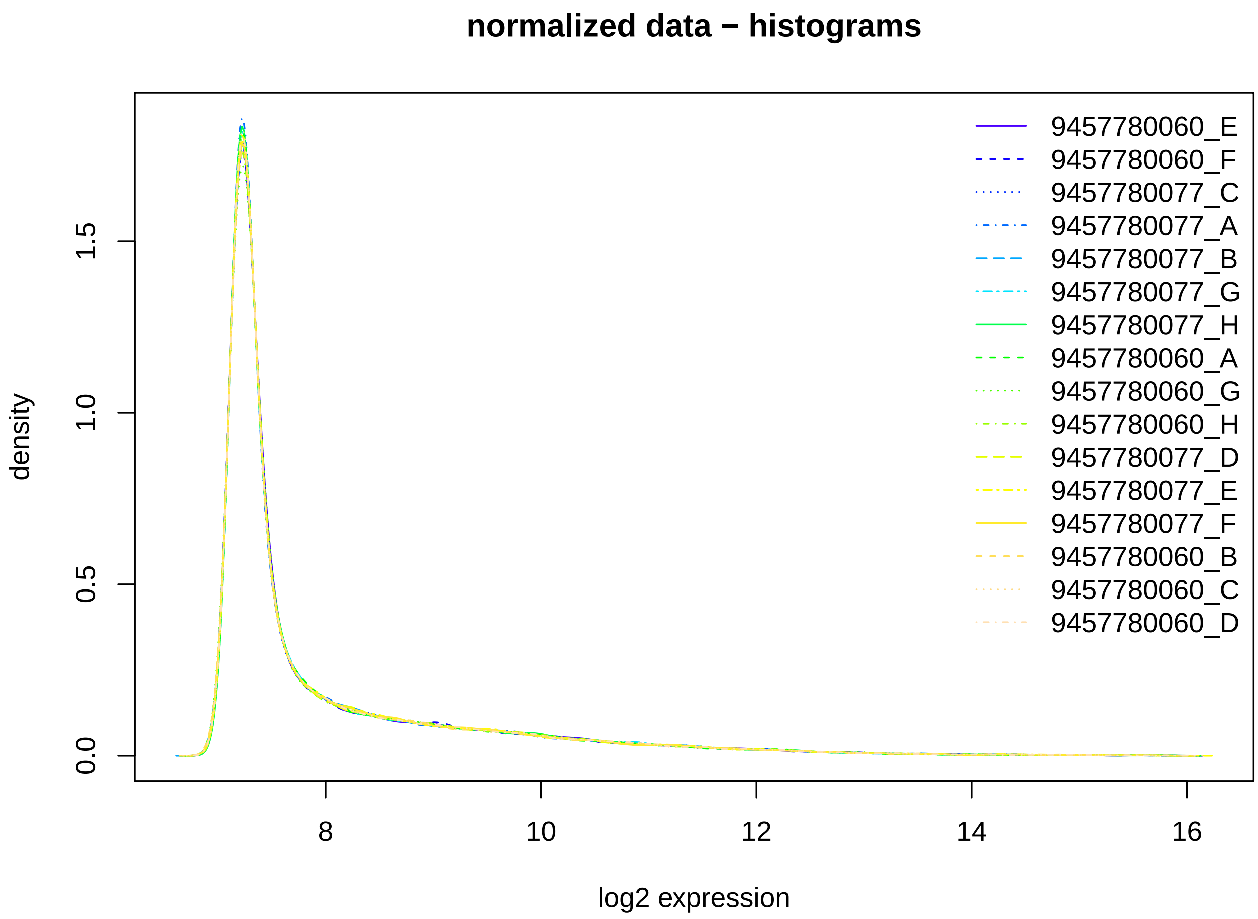

Figure 19: signal distribution for WT/PHIL arrays after normalization

Exploratory analysis of *WT vs PHIL* exper

Figure 20 - hierarchical clustering

using hierarchical clustering to group samples based on similarity/disimilarity

```
distance <- dist(t(filtered.matrix),method="maximum")
clusters <- hclust(distance, method = "average")
plot(clusters, label = sampleLabels.PHIL, main="Trichinella in WT/PHIL mice")
```

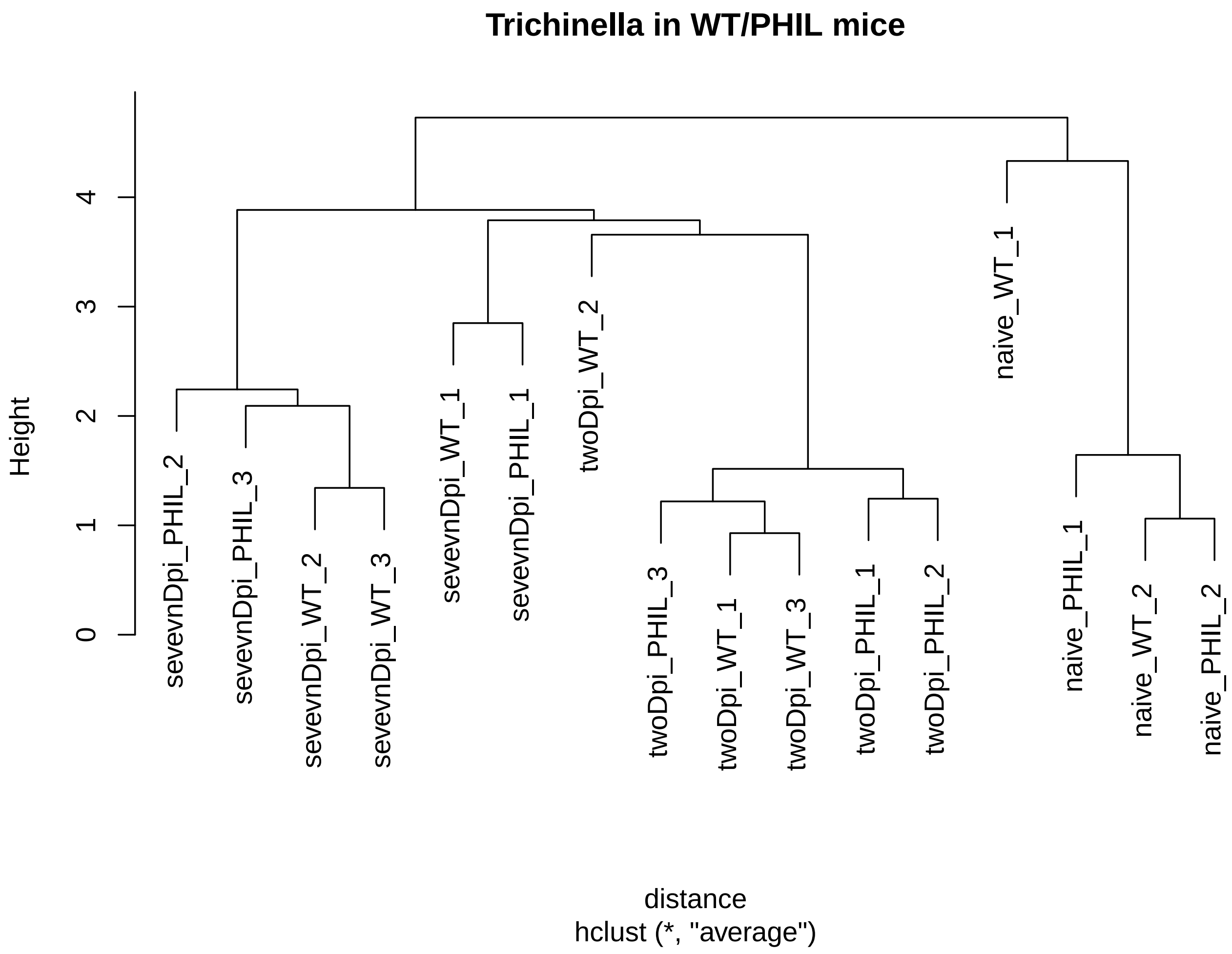

Figure 20: hierarchical clustering dendogram for WT/PHIL arrays

PCA: carrying out a Principle Component Analysis. Result shows that first two principle components account for 80% of the variation in the data

```
pca.res <- prcomp(t(filtered.matrix), scale.=F, retx=T)
ls(pca.res)
summary(pca.res)
head(pca.res$rotation)
head(pca.res$x)
#plot(pca.res, las=1)
```

```
pc.var<-pca.res$sdev^2
pc.per<-round(pc.var/sum(pc.var)*100, 1)
pc.per
```

Figure 21 - Principal component analysis

Lets see how each sample contributed to these first two principle components

```
data.frame <- as.data.frame(pca.res$x)
ggplot(data.frame, aes(x=PC1, y=PC2, colour=factor(groups.PHIL))) +
  geom_point(size=5) +
  theme(legend.position="right")
```

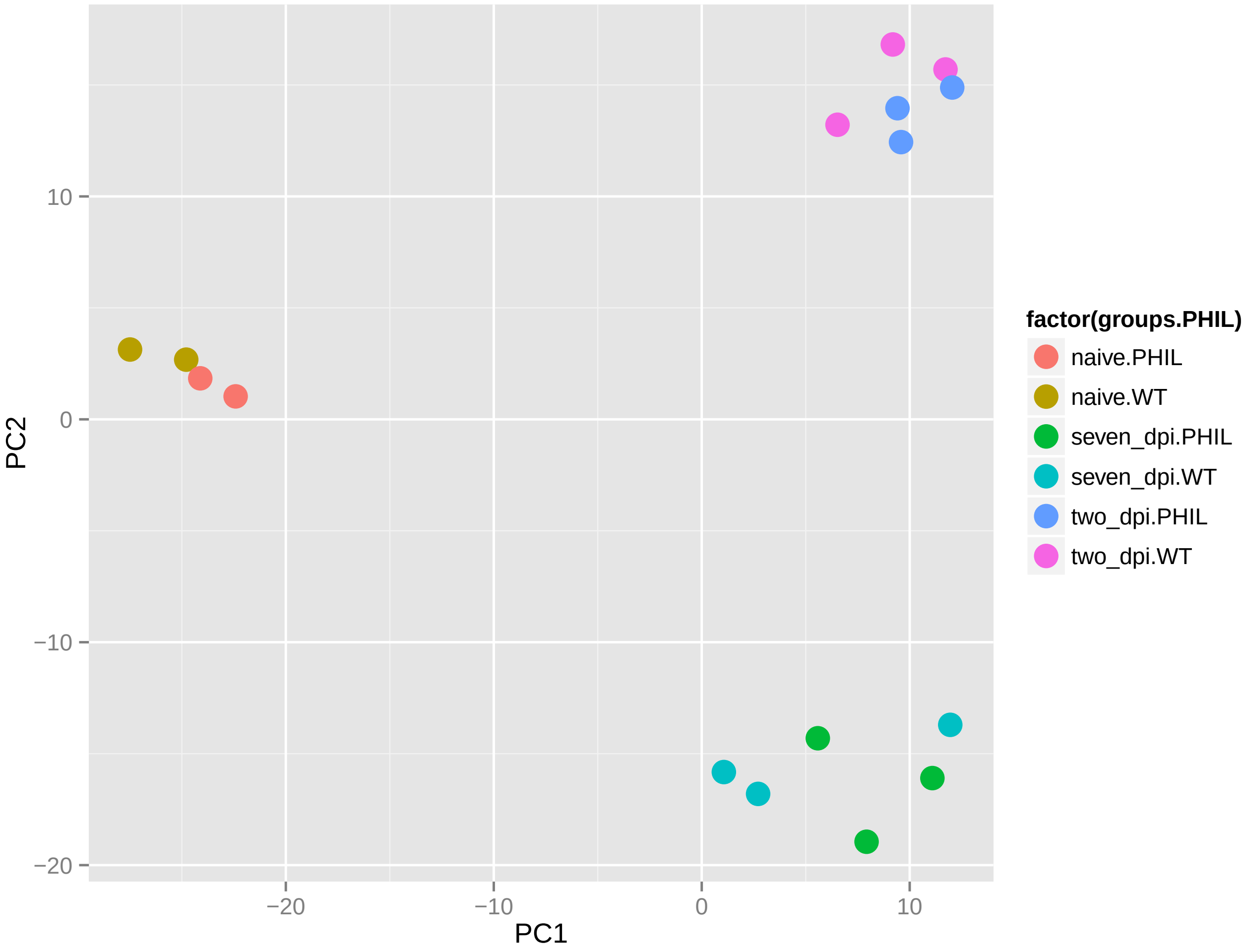

Figure 21: PCA plot for WT/PHIL data

Figure 22 - PCA 'small multiples' graph

```
melted <- cbind(groups.PHIL, melt(pca.res$x[,1:4]))
#look at your 'melted' data
ggplot(melted) +
  geom_bar(aes(x=Var1, y=value, fill=groups.PHIL), stat="identity") +
  facet_wrap(~Var2)
```

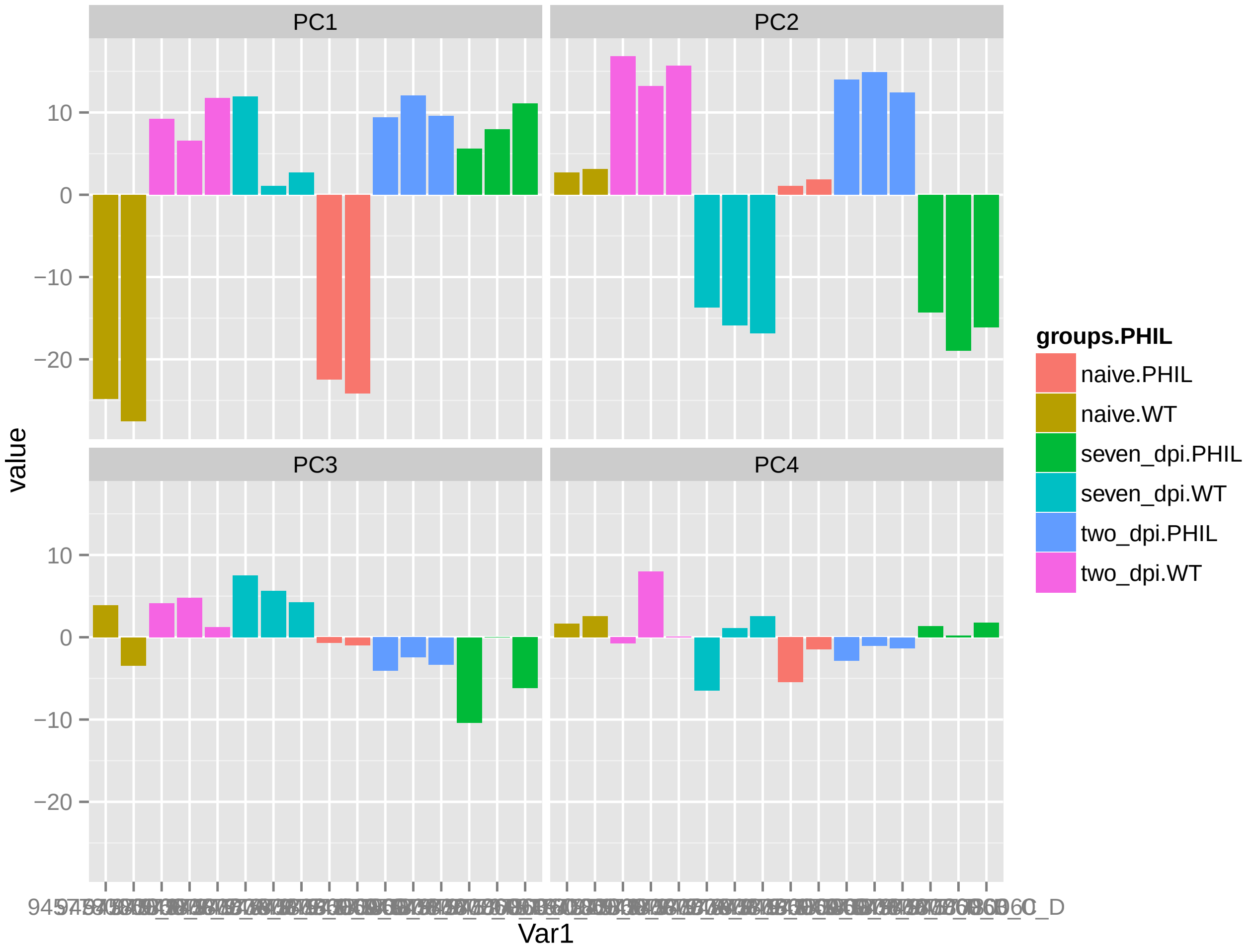

Figure 22: PCA 'small multiples' plot for WT/PHIL data

Setting up experimental design: This is a critical point in our analysis where we can begin to ask specific questions about which, if any, genes were differentially expressed. To do this, we must first define our questions in the form of a model matrix

```
design.PHIL <- model.matrix(~0+groups.PHIL)
colnames(design.PHIL) <- levels(groups.PHIL)
design.PHIL
```

Identification of Differentially Expressed Genes in *WT vs PHIL* exper

Fitting linear model to data and set-up contrast matrix

```
fit.PHIL <- lmFit(filtered.matrix, design.PHIL)
#add annotation into the linear model
fit.PHIL$genes$Symbol <- getSYMBOL(probeList, "lumiMouseAll.db")
fit.PHIL$genes$Entrez <- getEG(probeList, "lumiMouseAll.db")
# set up a contrast matrix based on the pairwise comparisons of interest
contrast.matrix.WTvsPHIL <- makeContrasts(latePHIL = seven_dpi.PHIL - seven_dpi.WT, levels=design.PHIL)
fits.PHIL <- contrasts.fit(fit.PHIL, contrast.matrix.WTvsPHIL)
ebFit.PHIL <- eBayes(fits.PHIL)
```

Table 4 - top 20 DEGs; WT vs PHIL at 7dpi

Given that our hierarchical cluster dendogram of all the samples showed pretty good separation of the three treatment groups, let's start by just asking to see the top 20 genes most significantly different between WT and PHIL mice at 7dpi

```
# use topTable function to take a look at the top most differentially expressed genes between
probeset.list <- topTable(ebFit.PHIL, adjust ="BH", coef=1, number=20, sort.by="logFC")
row.names(probeset.list) <- probeset.list[,1]
probeset.list <- probeset.list[,c(2,3,7)]
knitr::kable(probeset.list, caption="top 20 DEGs; WT vs PHIL at 7dpi")
```

Table 4: top 20 DEGs; WT vs PHIL at 7dpi

	ID.Entrez	logFC	adj.P.Val
Igtp	16145	1.1883081	0.0415676
Cxcl9	17329	1.1147505	0.0540617
Serpina3g	20715	1.0937197	0.0367468
Xlr4a	434794	0.9788597	0.1062014
Grb10	14783	-0.9644988	0.3007571
Gbp2	14469	0.9548508	0.0559801
Cxcl10	15945	0.8937234	0.0597212
Cd274	60533	0.8926974	0.1022803
Irgm2	54396	0.8781577	0.0559801
Mzb1	69816	0.8260500	0.0691635
Gbp3	55932	0.8184191	0.0346912
Irgm1	15944	0.8034547	0.1089648
Vegfa	22339	-0.7562695	0.0367468
Serpina3f	238393	0.7494373	0.0346912
Cd8b1	12526	0.7405668	0.0167416
Fcgr4	246256	0.7394661	0.1222695
H2-T23	15040	0.7368378	0.0484420
Upk3b	100647	0.7343783	0.0540617
Nt5e	23959	-0.7202767	0.0013216
Slc25a3	18674	-0.7170385	0.0201627

pull out these differentially expressed genes

```
diffProbes <- which(results[,1] !=0)
diffSymbols <- fit.PHIL$genes$Symbol[results[,1] !=0]
diffEntrez <- fit.PHIL$genes$Entrez[results[,1] !=0]
```

```
#convert to an expressionSet object
myEset <- new("ExpressionSet", exprs = filtered.matrix)
#link the eset to annotation data
annotation(myEset) <- "lumiMouseAll.db"
diffData <- myEset[results[,1] !=0]
#pull the expression data back out of the eset object
diffData <- exprs(diffData)
dim(diffData)
write.table(cbind(diffSymbols, diffEntrez, diffData),
            "diffGenes_PHIL.vs.WT.xls", sep="\t", quote=FALSE)
```

average biological replicates so we can make the least cluttered heatmap possible

```
head(diffData)
colnames(diffData) <- groups.PHIL
rownames(diffData) <- diffSymbols
head(diffData)
diffData.AVG <- avearrays(diffData)
head(diffData.AVG)
```

Clustering of differentially expressed genes from *WT vs PHIL* exper

Figure 23 - heatmap for WT/PHIL data

make heatmap from the 275 differentially expressed genes that were identified above.

```
#cluster rows by pearson correlation
hr <- hclust(as.dist(1-cor(t(diffData.AVG), method="pearson")), method="average")
#cluster columns by spearman correlation
hc <- hclust(as.dist(1-cor(diffData.AVG, method="spearman")), method="complete")
# Cut the resulting tree and create color vector for clusters.
mycl <- cutree(hr, k=6)
mycolhc <- rainbow(length(unique(mycl)), start=0.1, end=0.9)
mycolhc <- mycolhc[as.vector(mycl)]
myheatcol <- greenred(75)
#plot the hclust results as a heatmap
heatmap.2(diffData.AVG, Rowv=as.dendrogram(hr),
          Colv=NA, col=myheatcol, scale="row", labRow=NA,
          density.info="none", trace="none", RowSideColors=mycolhc,
          cexRow=1.5, cexCol=1, key=T, keysize=1, margins=c(10,30))
```

Figure 24 - selected genes from comparison of WT vs PHIL mice

10 genes were selected from a total of 275 differentially expressed genes above. *This plot corresponds to Figure 7c in the manuscript*

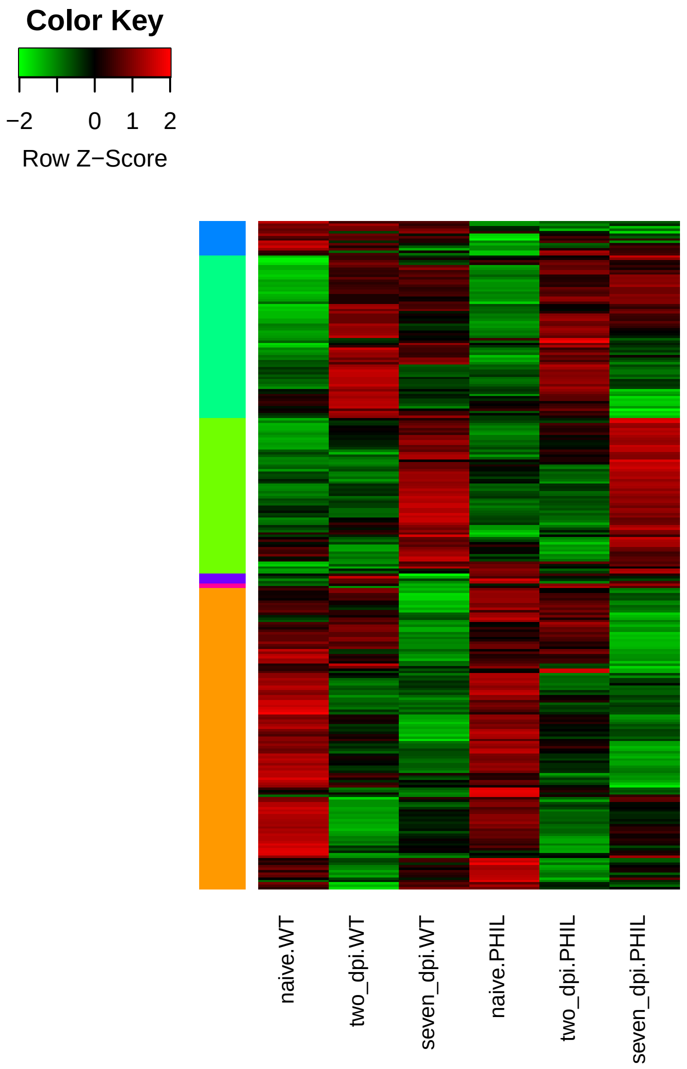

Figure 23: heatmap of 275 DEGs between WT and PHIL at 7dpi

```
#print out row labels in same order as shown in the heatmap
mySelected_PHIL <- read.delim("WTvsPHIL_selected.txt", sep="\t", stringsAsFactors = FALSE, header=TRUE,
mySelected_PHIL.matrix <- as.matrix(mySelected_PHIL)
heatmap.2(mySelected_PHIL.matrix,
          Rowv=NA, Colv=NA,
          col=myheatcol, scale="row",
          density.info="none", trace="none",
          labCol=NA, cexRow=1.5, cexCol=1,
          key=T, keysize=1, margins=c(10,30))
```

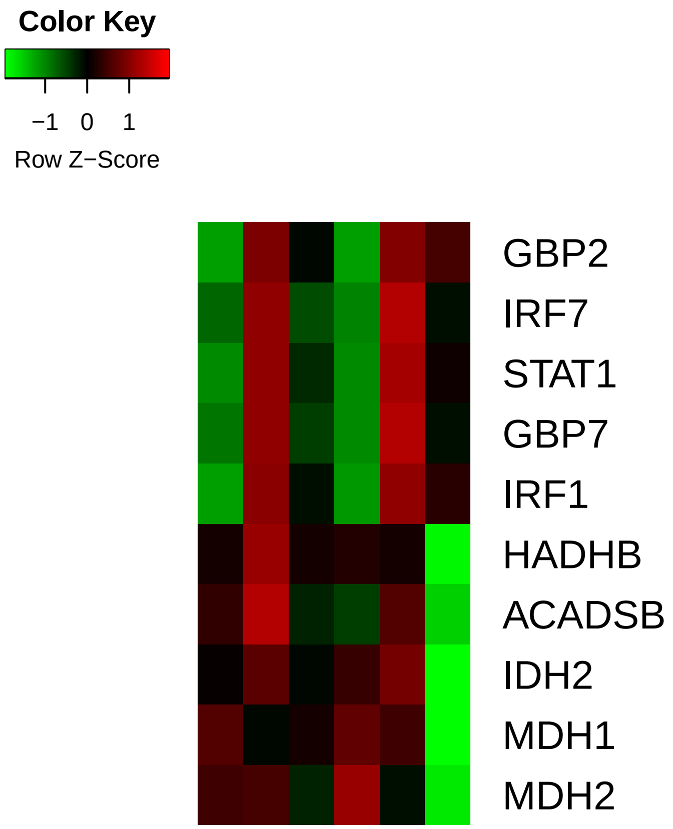

Figure 24: Ten selected genes from the WT vs PHIL heatmap shown above (corresponds to Figure 7c in the manuscript)

Session Info

Session Info: R version 3.1.3 (2015-03-09) Platform: x86_64-apple-darwin13.4.0 (64-bit) Running under: OS X 10.10.3 (Yosemite)

locale: [1] en_US.UTF-8/en_US.UTF-8/en_US.UTF-8/C/en_US.UTF-8/en_US.UTF-8

attached base packages: [1] stats4 parallel stats graphics grDevices utils datasets [8] methods base

other attached packages: [1] knitr_1.10.5 rmarkdown_0.7
[3] dplyr_0.4.1 reshape2_1.4.1
[5] annotate_1.44.0 XML_3.98-1.2
[7] limma_3.22.7 genefilter_1.48.1

[9] ggplot2_1.0.1 gplots_2.17.0
[11] RColorBrewer_1.1-2 lumiMouseAll.db_1.22.0
[13] org.Mm.eg.db_3.0.0 lumiMouseIDMapping_1.10.0 [15] RSQLite_1.0.0 DBI_0.3.1
[17] AnnotationDbi_1.28.2 GenomeInfoDb_1.2.5
[19] IRanges_2.0.1 S4Vectors_0.4.0
[21] lumi_2.18.0 Biobase_2.26.0
[23] BiocGenerics_0.12.1

loaded via a namespace (and not attached): [1] affy_1.44.0 affyio_1.34.0
[3] assertthat_0.1 base64_1.1
[5] base64enc_0.1-2 BatchJobs_1.6
[7] BBmisc_1.9 beanplot_1.2
[9] BiocInstaller_1.16.5 BiocParallel_1.0.3
[11] biomaRt_2.22.0 Biostrings_2.34.1
[13] bitops_1.0-6 brew_1.0-6
[15] bumphunter_1.6.0 caTools_1.17.1
[17] checkmate_1.5.3 codetools_0.2-11
[19] colorspace_1.2-6 digest_0.6.8
[21] doRNG_1.6 evaluate_0.7
[23] fail_1.2 foreach_1.4.2
[25] formatR_1.2 gdata_2.16.1
[27] GenomicAlignments_1.2.2 GenomicFeatures_1.18.7 [29] GenomicRanges_1.18.4 grid_3.1.3
[31] gtable_0.1.2 gtools_3.4.2
[33] highr_0.5 htmltools_0.2.6
[35] illuminaio_0.8.0 iterators_1.0.7
[37] KernSmooth_2.23-14 labeling_0.3
[39] lattice_0.20-31 locfit_1.5-9.1
[41] magrittr_1.5 MASS_7.3-40
[43] Matrix_1.2-1 matrixStats_0.14.0
[45] mclust_5.0.1 methylumi_2.12.0
[47] mgcv_1.8-6 minfi_1.12.0
[49] multtest_2.22.0 munsell_0.4.2
[51] nleqslv_2.8 nlme_3.1-120
[53] nor1mix_1.2-0 pkgmaker_0.22
[55] plyr_1.8.3 preprocessCore_1.28.0
[57] proto_0.3-10 quadprog_1.5-5
[59] Rcpp_0.11.6 RCurl_1.95-4.7
[61] registry_0.2 reshape_0.8.5
[63] rngtools_1.2.4 Rsamtools_1.18.3
[65] rtracklayer_1.26.3 scales_0.2.5
[67] sendmailR_1.2-1 siggenes_1.40.0
[69] splines_3.1.3 stringi_0.5-5
[71] stringr_1.0.0 survival_2.38-1
[73] tools_3.1.3 xtable_1.7-4
[75] XVector_0.6.0 yaml_2.1.13
[77] zlibbioc_1.12.0
